# Efficacy and safety of Xiao’er Fengre Qing oral liquid *versus* Oseltamivir in treating pediatric influenza (wind-heat invading the defense syndrome): a multicenter, randomized, non-inferiority trial

**DOI:** 10.3389/fphar.2025.1584003

**Published:** 2025-05-22

**Authors:** Shengxuan Guo, Xinmin Li, Yuejie Zheng, Chengliang Zhong, Lei Xiong, Xi Ming, Ying Ding, Yongbin Yan, Baoqing Zhang, Peng Zhou, Zhou Fu, Jun Wang, Xuefeng Wang, Junhong Wang, Jinghua Yang, Yanxia Liu, Jianxin Cai, Lihua Ning, Xiaohong Liu, Hang Zhu, Linlin Gai, Pingding Liu, Dahong Sun, Taizhong Wang, Xiaojiao Li, Xinhua Tian, Junguang Zhang, Wenda Guan, Yupin Li, Xueming Li, Junfeng Liu, Nanyue Kuang, Ling Lu, Tongxun Gao, Haodong Liang, Kunling Shen, Rong Ma

**Affiliations:** ^1^ Department of Clinical Trial Center, First Teaching Hospital of Tianjin University of Traditional Chinese Medicine, National Clinical Research Center for Chinese Medicine Acupuncture and Moxibustion, Tianjin, China; ^2^ Department of Pediatrics, First Teaching Hospital of Tianjin University of Traditional Chinese Medicine, National Clinical Research Center for Chinese Medicine Acupuncture and Moxibustion, Tianjin, China; ^3^ Department of Respiratory Medicine, Shenzhen Children’s Hospital, Shenzhen, China; ^4^ Department of Pediatrics, Yunnan Provincial Hospital of Traditional Chinese Medicine, Kunming, China; ^5^ Department of Pediatrics, The First Affiliated Hospital of Henan University of Traditional Chinese Medicine, Zhengzhou, China; ^6^ Department of Pediatrics, Affiliated Hospital of Shandong University of Traditional Chinese Medicine, Jinan, China; ^7^ Department of Respiratory Medicine, Children’s Hospital of Chongqing Medical University, Chongqing, China; ^8^ Department of Pediatrics, The Affiliated Hospital of Xuzhou Medical University, Xuzhou, China; ^9^ Department of Pediatrics, Affiliated Hospital of Liaoning University of Traditional Chinese Medicine, Shenyang, China; ^10^ Department of Pediatrics, Dongzhimen Hospital, Beijing University of Chinese Medicine, Beijing, China; ^11^ Department of Pediatrics, Guangdong Provincial Hospital of Chinese Medicine, Guangdong, China; ^12^ Department of Pediatrics, Shijiazhuang Maternal and Child Healthcare Hospital, Shijiazhuang, China; ^13^ Department of Pediatrics, Wuhan Hospital of Traditional Chinese Medicine, Wuhan, China; ^14^ Department of Pediatrics, The Fourth Hospital of Baotou, Baotou, China; ^15^ Department of Pediatrics, Baoji Maternal and Child Healthcare Hospital, Baoji, China; ^16^ Department of Pediatrics, 215 Hospital of Shaanxi Nuclear Industry, Xianyang, China; ^17^ Department of Pediatrics, Ansteel Group General Hospital, Anshan, China; ^18^ Department of Pediatrics, Weinan Maternal and Child Health Hospital, Weinan, China; ^19^ Department of Pediatrics, The Affiliated Qingdao Third People’s Hospital of Qingdao University, Qingdao, China; ^20^ Department of Pediatrics, Hegang People’s Hospital, Hegang, China; ^21^ Department of Pediatrics, Yinchuan Traditional Chinese Medicine Hospital, Yinchuan, China; ^22^ Department of Pediatrics, Zhongwei People’s Hospital, Zhongwei, China; ^23^ Department of Pediatrics, Fengfeng General Hospital of North China Medical Health Group, Handan, China; ^24^ Department of Pediatrics, Mudanjiang Traditional Chinese Medicine Hospital, Mudanjiang, China; ^25^ Department of Pediatrics, Xi’an Daxing Hospital, Xi’an, China; ^26^ Department of Pediatrics, Handan Hospital of Traditional Chinese Medicine, Handan, China; ^27^ Department of Pediatrics, Handan First Hospital, Handan, China; ^28^ Department of Pediatrics, Xinjiang Production and Construction Corps Hospital, Wulumuqi, China; ^29^ Department of Pediatrics, Zaozhuang Municipal Hospital, Zaozhuang, China

**Keywords:** Xiao’er Fengre Qing oral Liquid, influenza, pediatrics, wind-heat invading the defense Syndrome, traditional Chinese medicine, multicenter, randomized controlled trial

## Abstract

**Background:**

Xiao’er Fengre Qing Oral Liquid (XFQOL) is developed based on the classical traditional Chinese medicinal formula *Yinqiao Powder*. Compared to the original formulation, XFQOL exhibits enhanced heat-clearing, detoxification, and fever reduction, which can effectively address the common complications associated with influenza in children and is well-suited for pediatric use. However, there is currently a lack of high-quality evidence from clinical trials to support its efficacy and safety in clinical applications.

**Objective:**

This study aimed to investigate the efficacy and safety of XFQOL compared with Oseltamivir in pediatric influenza.

**Methods:**

A multicenter, block-randomized, double-blind, double-dummy, positive-drug-controlled, non-Inferiority clinical trial design was conducted. The study plans to enroll 420 pediatric participants, with 210 in each group. The experimental group will receive XFQOL with an Oseltamivir granules placebo, and the control group will receive Oseltamivir granules with a XFQOL placebo for 5 days, followed by a 2-day post-treatment observation. The primary endpoint was clinical recovery time, while secondary endpoints included complete fever resolution time, the area under the curve (AUC) of Canadian Acute Respiratory Illness and Flu Scale (CARIFS) symptom dimension Score over time, Traditional Chinese Medicine (TCM) syndrome efficacy, disappearance rates for individual symptoms, incidences of complications and severe and critical influenza, the usage of acetaminophen, and viral negative conversion rate. Safety evaluation focused on adverse events (AE) and adverse drug reactions (ADR).

**Results:**

A total of 418 participants were included in the Full Analysis Set, with 208 in the experimental group and 210 in the control group. Baseline characteristics were comparable between the groups. The median time to clinical recovery was 3 days for both groups, with a hazard ratio and its 95% confidence interval (experimental group/control group) of 1.115 (95% CI: 0.912–1.363). Non-inferiority testing demonstrated that the experimental group was not inferior to the control group. Subgroup analyses (positive for RT-PCR influenza, positive for RT-PCR influenza A, positive for RT-PCR influenza B) yielded results consistent with the primary endpoint. The median time to complete fever resolution was 32 h in both groups, with no statistically significant difference (*P* = 0.407). There were no statistically significant differences in the AUC of CARIFS symptom scores over time between the groups (*P* = 0.211). No significant differences were observed between the groups in the efficacy rates of TCM syndromes of Wind-Heat Invading the Defense Syndrome (*P* = 0.076) and Fright-complicated Syndrome (*P* = 0.168); however, significant differences were found in Phlegm-complicated Syndrome (*P* = 0.008) and Food-stagnation-complicated Syndrome (*P* = 0.024). The disappearance rates for individual symptoms, such as red and swollen pharynx, cough, copious sputum or audible phlegm sounds in the throat, and lack of appetite, showed statistically significant differences between the groups (*P* < 0.05), while no significant differences were observed for other symptoms. No statistically significant differences were observed between the experimental and control groups in the incidence of complications and severe and critical influenza, the usage of acetaminophen, and viral negative conversion rate (*P* > 0.05). The incidence rates of AE (*P* = 0.885) and ADR (*P* = 0.685) were comparable between the two groups, with no statistically significant differences observed.

**Conclusion:**

The efficacy of XFQOL in treating pediatric influenza (Wind-Heat Invading the Defense Syndrome) is non-inferior to Oseltamivir with respect to clinical recovery time. Additionally, its effectiveness in terms of fever reduction, symptom alleviation, incidences of complications and severe and critical influenza, the usage of acetaminophen, and viral negative conversion rate is comparable to that of Oseltamivir. Furthermore, it demonstrates good safety, suggesting its potential for clinical application.

**Clinical Trial Registration:**

clinicaltrials.gov, identifier ChiCTR2300076191.

## 1 Introduction

Influenza is an acute respiratory infectious disease caused by influenza viruses, leading to seasonal epidemics and, occasionally, rare and unpredictable pandemics that impose a substantial burden on global healthcare systems annually. Children worldwide constitute the demographic most affected by influenza, exhibiting the highest infection and morbidity rates, with younger children particularly susceptible to severe outcomes (Uyeki et al., 2022). Recent epidemiological estimates reveal that influenza affects approximately 109.5 million children under 5 years old each year globally, leading to 870,000 hospitalizations and 34,800 deaths (Wang et al., 2020). Surveillance data from mainland China reveal that 65.41% of influenza cases occur in individuals under 15 years old, highlighting children and students as key populations for prevention and control (Yang et al., 2018). Among the various syndrome types of pediatric influenza, “Wind-Heat Invading the Defense Syndrome” is the most common, comprising approximately 50% of cases (Zhang et al., 2023). Neuraminidase inhibitors remain the most widely endorsed antiviral agents in domestic and international guidelines, with Oseltamivir being the most extensively used drug for managing seasonal influenza and stockpiling during pandemics (Hurt and Kelly, 2016; Ma R. et al., 2024). A meta-analysis demonstrated that the prompt administration of Oseltamivir shortens illness duration in children by an average of 18 h compared to placebo (Malosh et al., 2018). However, the therapeutic efficacy of antiviral drugs is frequently hindered by viral mutations and escalating resistance attributable to selective drug pressure (Sarker et al., 2022). The WHO Influenza Guidelines (2024) reemphasize the challenge of antiviral resistance, noting that all recommended drugs exhibit varying levels of resistance. Even newer antivirals such as Baloxavir Marboxil are not immune to resistance (Centers for Disease Control and Prevention, 2024). The problem of drug resistance is particularly pronounced in pediatric populations (Kiso et al., 2004). Furthermore, limitations encompass suboptimal clinical efficacy, cost-effectiveness concerns, potential adverse effects (e.g., nausea and vomiting), and insufficient high-quality evidence from rigorous clinical studies (Malosh et al., 2018). Therefore, exploring alternative or adjunctive treatment methods that can effectively alleviate symptoms while maintaining a high level of safety holds significant clinical value.

Chinese Herbal Medicine exerts multi-targeted and multi-layered therapeutic effects, demonstrating partial broad-spectrum antiviral activity (Ma R. et al., 2024). They not only suppress viral replication directly but also regulate immune responses, mitigate complications. Xiao’er Fengre Qing Oral Liquid (XFQOL) was approved by the Chinese National Medical Products Administration in 2020. It is characterized by its ability to release exterior syndrome with pungent-cool medicinals, clear heat and remove toxins, relieve cough and soothe the throat, making it suitable for pediatric influenza and acute upper respiratory tract infections (Wang M. et al., 2024). This formula is derived from Yinqiao Powder, a classic Traditional Chinese Medicine (TCM) prescription traditionally used to treat early-stage febrile diseases characterized by wind-heat syndrome. The formulation of XFQOL is tailored to the unique physiological characteristics of children, characterized by “insufficient pulmonary and splenic functions but surplus cardiac and hepatic activity,” as well as the pathological features of common cold in children, including tendencies toward phlegm retention, food stagnation, and fright. This medication not only possesses the heat-clearing, detoxifying, and antipyretic effects of *Yinqiao Powder*, but also provides phlegm-resolving, digestion-promoting, and fright-calming properties. However, there is currently a lack of high-quality evidence from clinical trials to support its efficacy and safety in clinical applications. This study aimed to investigate the efficacy and safety of XFQOL compared with Oseltamivir in pediatric influenza.

## 2 Methods

### 2.1 Study design

This study employed a multicenter, block-randomized, double-blind, double-dummy, positive drug parallel-controlled non-inferiority design. The blinding procedure was performed in two stages: the first stage assigned groups 1 and 2, and the second stage designated group 1 as the experimental group and group 2 as the control group. The statistician employed a block randomization method to allocate participants at a 1:1 ratio between the experimental and control groups. The random allocation sequence was generated using the PROC PLAN procedure in SAS V9.4 software, with a predefined seed number to generate random numbers and assign corresponding treatment interventions. The drug blinding and preparation of emergency unblinding letters were performed by personnel not involved in the clinical trial. The blinding codes were stored in duplicate, with one copy at the research site and the other with the sponsor. All drugs were assigned unique identifiers, which remained unchanged throughout the study. The trial was spearheaded by the First Teaching Hospital of Tianjin University of Traditional Chinese Medicine and conducted simultaneously at 29 tertiary medical centers across China. The study obtained ethical approval from the Ethics Committee of the First Teaching Hospital of Tianjin University of Traditional Chinese Medicine (Approval Number: TYLL2023(Y)017) and was duly registered with the Chinese Clinical Trial Registry (ChiCTR2300076191, www.chictr.org.cn). Written informed consent was obtained from all participants. The trial adhered rigorously to the ethical standards outlined in the World Medical Association’s Declaration of Helsinki and the International Conference on Harmonisation’s Guidelines for Good Clinical Practice.

### 2.2 Study medication

Xiao’er Fengre Qing Oral Liquid (National Medical Products Administration, National Drug Approval Number Z19990012, manufactured by Handan Pharmaceutical Co., Ltd., with a specification of 10 mL × 10 bottles/box) is a brownish-red to brown liquid with a sweet and slightly bitter taste, and possesses the properties of releasing exterior syndrome with pungent-cool medicinals, clearing heat and removing toxins, relieving cough and soothing the throat. Xiao’er Fengreqing Oral Liquid is a standardized TCM formulation composed of 20 species of Chinese materia medica, including Honeysuckle *(Lonicera japonica (Thunb.)*, the dried flower buds or early-opening flowers of the *L. japonica* plant of the Caprifoliaceae family), Forsythia (*Forsythia suspensa (Thunb.) Vahl*, the dried fruits of Forsythia suspensa from the Oleaceae family), Isatis (*Isatis indigotica Fort.*, the dried roots of the Isatis indigotica plant from the Cruciferae family), Mint (*Mentha haplocalyx Briq.*, the dried aerial parts of the Mentha haplocalyx plant from the Lamiaceae family), Bupleurum (*Bupleurum chinense DC*., the dried roots of the Bupleurum chinense plant from the Apiaceae family), Arctium (*Arctium lappa L.*, the dried mature fruits of the Arctium lappa plant from the Asteraceae family), Schizonepeta (*Schizonepeta tenuisfolia Briq*., the dried flower spikes of the Schizonepeta tenuisfolia plant from the Lamiaceae family), Gypsum (a sulfate mineral, primarily composed of calcium sulfate dihydrate (CaSO4 ⋅ 2H2O)), Baikal Skullcap (*Scutellaria baicalensis Georgi.*, the dried roots of the Scutellaria baicalensis plant from the Lamiaceae family), Gardenia (*Gardenia jasminoides Ellis*, the dried mature fruit of the Gardenia jasminoides plant from the Rubiaceae family), Platycodon (*Platycodon grandiflorum (Jacq.) A. DC.*, the dried roots of the Platycodon grandiflorum plant from the Campanulaceae family), Red Peony (*Paeonia lactiflora Pall.* or *Paeonia veitchii Lynch*, the dried roots of the Peony plant from the Ranunculaceae family), Reed Root (*Phragmites communis Trin*., the dried rhizomes of the Reed plant from the Poaceae family), Bitter Apricot Kernel (*Prunus armeniaca L.* var. *ansu Maxim.*, *Prunus sibirica L.*, *Prunus mandshurica (Maxim.) Koehne*, or *P. armeniaca L*., the dried mature seeds of the Prunus species from the Rosaceae family), Lophatherum (*Lophatherum gracile Brongn*., the dried stems and leaves of the Lophatherum gracile plant from the Poaceae family), Immature Orange Peel (*Citrus aurantium L.*, the dried immature fruits of the Citrus aurantium species from the Rutaceae family), Six Shenqu (a traditional fermented medicament prepared by mixing *Polygonum hydropiper*, *Artemisia annua*, *P. armeniaca kernel* with wheat flour, followed by microbial fermentation), Silkworm (*Bombyx mori Linnaeus*, the dried bodies of larvae infected with the white muscardine fungus, *Beauveria bassiana (Bals.) Vuillant*, from the family Bombycidae), Siler (*Saposhnikovia divaricata (Turcz.) Schischk.*, the dried roots of the Siler plant from the Apiaceae family), and Licorice (*Glycyrrhiza uralensis Fisch.*, *Glycyrrhiza inflata Bat.*, or *Glycyrrhiza glabra L.*, the dried roots and rhizomes of the Glycyrrhiza plant from the Fabaceae family).

XFQOL is processed through extraction, concentration and purification processes, and its quality complies with the national drug standards formulated by the National Medical Products Administration. Identification of reference medicinal materials, including arctiin, baicalin, geniposide, and forsythin, was conducted using thin-layer chromatography, and the quantification of these marker compounds was performed using high-performance liquid chromatography (HPLC) for enhanced analytical precision. Each 1 mL of XFQOL must contain not less than 0.70 mg of geniposide (C17H24O10), in strict accordance with the relevant mixture specifications outlined in the Chinese Pharmacopoeia (2020 edition, General Rule 0181), ensuring regulatory compliance and standardization (Heinrich et al., 2020). The characteristic chromatogram of XFQOL is provided in Supplementary Material S2. The product inspection reports of the study medication and the placebo are shown in Supplementary Material S3, 4.

The XFQOL placebo was formulated by diluting XFQOL, whereas the Oseltamivir placebo was mainly composed of granulated sugar, sucrose, and ethanol. The detailed preparation procedures for the placebo are detailedly outlined in Supplementary Material S1. XFQOL, Oseltamivir, and their respective placebos were matched in appearance, odor, taste and usage. Both investigational drugs and matching placebos were supplied by Handan Pharmaceutical Co., Ltd.

### 2.3 Participants

#### 2.3.1 Diagnostic criteria

The diagnostic criteria were formulated based on the *Guidelines for diagnosis and treatment of influenza* (2020), with an emphasis on clinical manifestations, epidemiological history, and etiological examination (General Office of the National Health Commission and Office of the National Administration of Traditional Chinese Medicine, 2021) (Supplementary Material S1).

The TCM syndrome differentiation criteria were developed referencing the *Guideline on the Design and Evaluation of Clinical Trials for Chinese Medicine in Common Pediatric Disease: Influenza* (Hu, 2021), *the Guidelines of prevention and treatment of children’s influenza A (*H1N1*) with TCM* (Ma et al., 2010), and the *Guideline for TCM pediatrics clinical diagnosis and treatment (2012)* (China Association of Chinese Medicine. Guideline for TCM pediatrics clinical diagnosis and treatment, 2012). The detailed diagnostic criteria for Wind-Heat Invading the Defense Syndrome, Phlegm-complicated Syndrome, Food-stagnation-complicated Syndrome, and Fright-complicated Syndrome are presented in the Supplementary Material S1.

#### 2.3.2 Inclusion criteria

(1) Meeting the diagnostic criteria for influenza.
(2) Meeting the diagnostic criteria for Wind-Heat Invading the Defense Syndrome, with or without accompanying syndromes.
(3) Positive rapid antigen test for influenza virus using nasopharyngeal or throat swab specimens.
(4) Age between 1 and 14 years (<15 years).
(5) Duration from fever onset to consultation ≤48 h, with a maximum axillary temperature ≥38.0°C.
(6) Informed consent must comply with regulations and be signed jointly by the legal guardian and the participating child (aged ≥8 years).

#### 2.3.3 Exclusion criteria

(1) Severe or critical cases of influenza.
(2) Patients with concurrent respiratory infections such as pharyngo-conjunctival fever, herpetic pharyngitis, or suppurative tonsillitis.
(3) Influenza complications already present, including but not limited to sinusitis, otitis media, bronchitis, pneumonia, myocarditis, encephalitis, or encephalopathy.
(4) Use of antiviral drugs within 48 h prior to the current consultation.
(5) Ongoing systemic treatment with corticosteroids or other immunosuppressive agents.
(6) History of epilepsy, febrile seizures, or recurrent respiratory infections.
(7) Presence of chronic underlying conditions, including respiratory, cardiac, renal, hepatic, hematologic, endocrine, neurological, or immunodeficiency disorders.
(8) Patients with glucose-6-phosphate dehydrogenase deficiency (G6PD deficiency).
(9) Allergy to any known component of the investigational drug.
(10) Patients deemed unsuitable for participation in the clinical trial by the investigator.

#### 2.3.4 Drop-out criteria

In cases where the condition worsens after medication, complications arise, influenza progresses to severe or critical stages, or other diseases occur, researchers may determine that the participating child is unsuitable for continued trial participation.

#### 2.3.5 Sample size calculation

Drawing on prior literature and clinical studies, the clinical recovery time for the control group was estimated at approximately 3 days (Heinonen et al., 2010; Treanor et al., 2000). A non-inferiority margin of Δ = 0.75 was set, with α = 0.025 (one-sided) and β = 0.2. Assuming a 1:1 allocation ratio, a sample size of 190 participants per group was estimated, totaling 380. Accounting for a 10% dropout rate, each group required 210 participants, leading to an overall total of 420 participants.

### 2.4 Investigators and researchers

All investigators and researchers involved in this trial held experienced attending physician qualifications or higher, possessed specialized expertise in clinical trials, and had successfully passed a qualification review. Prior to the initiation of the clinical trial, a standardized training program was implemented to ensure that all investigators and researchers acquired a thorough understanding of the study protocol and the specific implications of each indicator.

### 2.5 Interventions

(1) XFQOL (10 mL per bottle) was administered orally according to the following dosage schedule: 2.5–5 mL per dose for children aged 0–2 years, 5–10 mL per dose for children aged 3–5 years, and 7.5–15 mL per dose for children aged 6–14 years. The regimen consisted of four doses per day, with the suspension shaken well before use.
(2) Oseltamivir Phosphate Granules (15 mg per sachet; Kewei; approval No. H20080763) were administered orally based on body weight: 30 mg per dose for participants weighing ≤15 kg, 45 mg per dose for those weighing 15–23 kg, 60 mg per dose for those weighing 23–40 kg, and 75 mg per dose for those weighing >40 kg. This medication was given twice daily.

The experimental group received XFQOL and Oseltamivir Phosphate Granule placebo, while the control group received XFQOL placebo and Oseltamivir Phosphate Granules. Both groups underwent a 5-day treatment regimen, followed by a 2-day post-treatment follow-up (within 48 h after the final dose).

### 2.6 Concomitant medications

(1) Participants with axillary temperatures >38.5°C were administered acetaminophen at the investigator’s discretion to ensure participant safety. If the fever persisted, additional antipyretic and analgesic agents were allowed based on clinical necessity.
(2) In the presence of bacterial infection indications, appropriate antibiotic therapy will be administered.

### 2.7 Endpoints

#### 2.7.1 Primary endpoint

The primary endpoint of this trial was the time to clinical recovery (days). Clinical recovery was defined as a reduction in the patient’s body temperature to 37.2°C or lower following treatment, coupled with a symptom dimension score of ≤1 on the Canadian Acute Respiratory Illness and Flu Scale (CARIFS), sustained for more than 24 h.

#### 2.7.2 Secondary endpoints

(1) Complete Fever Resolution Time (hours): The time required for the patient’s body temperature to decrease to 37.2°C or lower and remain sustained for more than 24 h.
(2) Area under the Curve (AUC) of the CARIFS Symptom Dimension Score over Time.
(3) TCM Syndrome Scoring (Wind-Heat Invading the Defense Syndrome, Phlegm-complicated Syndrome, Food-stagnation-complicated Syndrome, and Fright-complicated Syndrome): TCM symptom grading and quantification standards are referenced from the Guideline on the *Design and Evaluation of Clinical Trials for Chinese Medicine in Common Pediatric Disease: Influenza* (Hu, 2021), the *G. for TCM pediatrics clinical diagnosis and treatment (2012)* (China Association of Chinese Medicine. Guideline for TCM pediatrics clinical diagnosis and treatment, 2012), and the *Guidelines for Clinical Research of New Traditional Chinese Medicines (Trial)* (Zheng, 2002). Primary and secondary symptoms are graded into four levels: none, mild, moderate, and severe. Primary symptoms (fever) are scored 0, 2, 4, and six points, respectively; secondary symptoms (headache, nasal congestion, rhinorrhea, sneezing, red and swollen pharynx, cough) are scored 0, 1, 2, and three points, respectively. Accompanied symptoms (copious sputum or audible phlegm sounds in the throat, distension and fullness in the epigastrium and abdomen, lack of appetite, foul breath, nausea and vomiting, acidic and putrid vomitus, acidic and malodorous stools, abdominal pain, diarrhea, constipation, fright and shrieking, restlessness and agitation) are scored on two levels: 0 and 1. Tongue and pulse manifestation are recorded but not scored. A valid TCM syndrome outcome is defined as a reduction in TCM syndrome score by ≥ 50%.
(4) Disappearance Rate of Individual Symptoms: Symptom disappearance is defined as a score of 0 for the symptom.
(5) Incidence of Complications and Severe and Critical Influenza: Complications include sinusitis, otitis media, bronchitis, and pneumonia, or hospitalization due to influenza. Definitions of severe and critical influenza are as outlined in the Diagnosis and Treatment Protocol for Influenza (2020) (General Office of the National Health Commission and Office of the National Administration of Traditional Chinese Medicine, 2021). (Supplementary Material S1).
(6) The Usage of acetaminophen.
(7) Viral Negative Conversion Rate: The proportion of baseline-positive participants achieving viral nucleic acid negativity at the end of treatment (5 ± 1 day following medication) in each group.

### 2.8 Safety analysis

Observation indicators: (i) incidence of clinical adverse events (AE)/adverse drug reactions (ADR); (ii) Vital Signs; (iii) Laboratory Tests: complete blood count, urinalysis, liver function, kidney function, electrocardiogram, and cardiac enzymes (optional). The incidence of AE/ADR will be the primary safety observation indicators.

### 2.9 Statistical methods

Statistical analysis was performed using SAS v9.4 software. Intention-to-treat analysis was performed utilizing the full analysis set (FAS). FAS was used for baseline data and efficacy analysis, including participants who were randomly assigned, received at least one dose of the study drug, and had at least one visit record. The safety set (SS) was used for safety analysis, including participants who received at least one dose of the study drug and had recorded safety data. Survival data were described by median survival time and interquartile range (IQR; Q1-Q3). Group comparisons were conducted using the log-rank test, and Kaplan-Meier curves were generated. Quantitative data were expressed as mean and standard deviation (SD), and between-group differences were assessed using t-tests. Qualitative data were presented as frequencies and percentages, with between-group comparisons performed using χ^2^ tests, Fisher’s exact tests, or Wilcoxon rank-sum tests. The primary endpoint was analyzed using a Cox proportional hazards regression model for non-inferiority testing, with hazard ratios (HR) and corresponding 95% confidence intervals (CI) computed between the experimental and control groups. If the lower bound of the 95% CI for the HR of the experimental group relative to the control group exceeded 0.75, non-inferiority was established. Apart from the non-inferiority test, which employed a one-sided significance level of α = 0.025, all other hypothesis tests were conducted using a two-sided approach, with a significance threshold of α = 0.05.

## 3 Results

### 3.1 Completion of the trial and data set division

**FIGURE 1:**
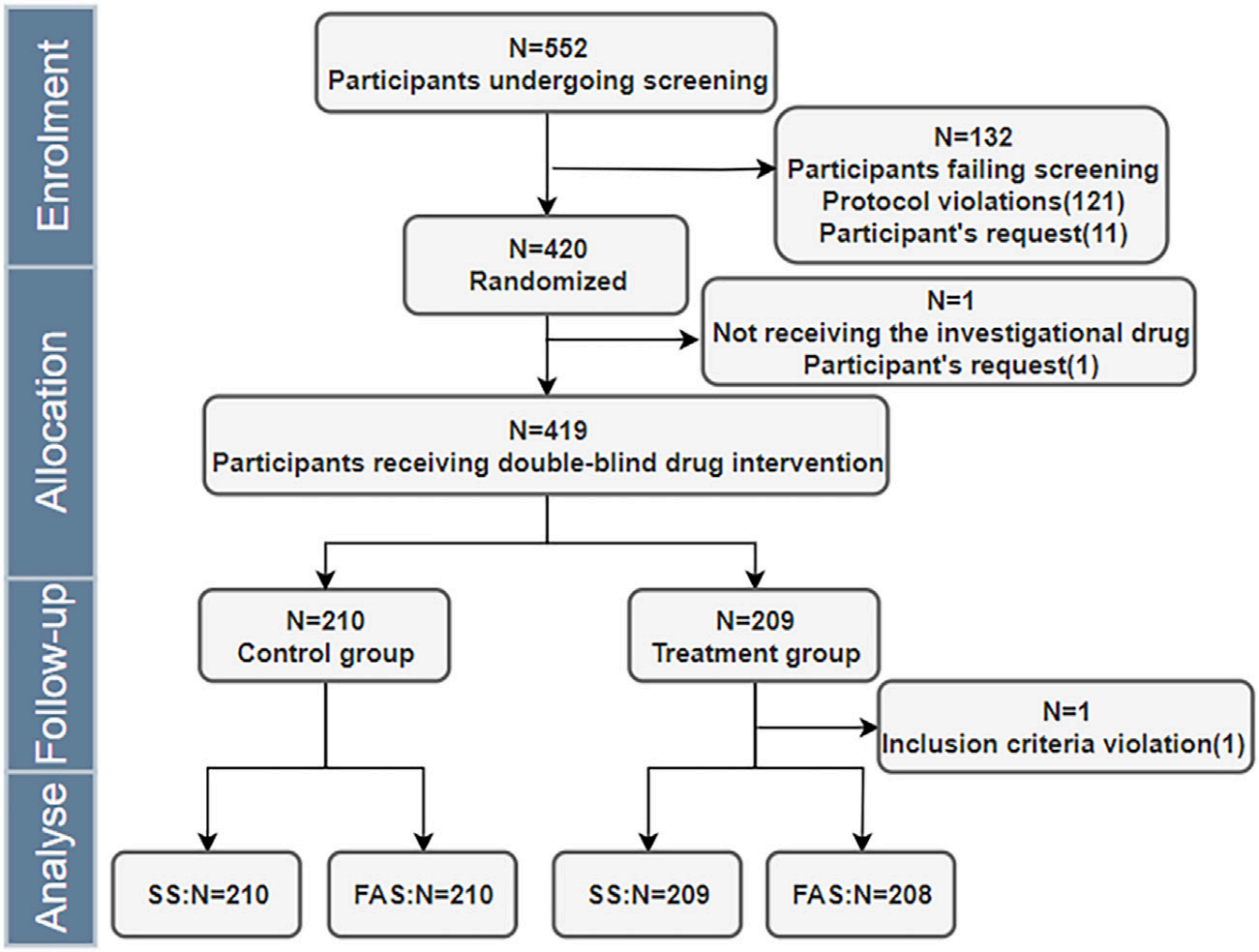
Completion of the Trial and Dataset Division. SS = safety set; FAS = full analysis set.

This trial was conducted from October 2023 to February 2024. A total of 420 participants, were enrolled and randomized at a 1:1 ratio into the experimental or control groups. FAS included a total of 418 participants, with two participants excluded from the experimental group (1 for not receiving the study drug and one for failing to meet inclusion criteria). SS included a total of 419 participants, with one participant excluded from the experimental group (for not receiving the study drug). Detailed procedures are illustrated in Figure 1.

### 3.2 Baseline characteristics

The analysis revealed no statistically significant differences between the two groups in demographic characteristics (sex, ethnicity, age, height, weight), disease conditions (disease duration, maximum temperature within 48 h before diagnosis, family history, allergy history, influenza vaccination within the past 12 months, and pre-treatment comorbidities and treatment history), accompanying syndromes (Phlegm-complicated Syndrome, Food-stagnation-complicated Syndrome, and Fright-complicated Syndrome), or influenza virus RT-PCR testing results (RT-PCR for influenza, RT-PCR for influenza A and RT-PCR for influenza B) (Table 1). Efficacy-related baseline indicators, including TCM syndrome scores, individual symptom scores (Supplementary Material S1), CARIFS scores and symptom dimension scores, also showed no significant differences between groups, confirming comparability.

**TABLE 1:** Comparison of baseline data between the two groups.

Variable	Description	Experimental group (n = 208)	Control group (n = 210)	*p-value*
*Demographics*				
Gender (n)	Male	113	118	0.702
	Female	95	92	
Ethnicity (n)	Han nationality	202	204	0.987
	Other ethnic groups	6	6	
Age (years)	Mean (SD)	7.490 ± 3.303	7.833 ± 3.055	0.271
Height (cm)	Mean (SD)	128.207 ± 22.810	131.010 ± 23.070	0.212
Weight (kg)	Mean (SD)	30.167 ± 13.720	31.607 ± 15.192	0.310
*Disease conditions*				
Disease duration (h)	Mean (SD)	24.889 ± 12.374	26.643 ± 12.613	0.152
Maximum temperature within 48 h before diagnosis (°C)	Mean (SD)	39.015 ± 0.526	38.991 ± 0.541	0.640
Family history (n)	No	208	209	1.000
	Yes	0	1	
Allergy history (n)	No	203	208	0.283
	Yes	5	2	
Influenza vaccination within the past 12 months (n)	No	204	207	0.723
	Yes	4	3	
Pre-treatment comorbidities (n)	No	195	194	0.582
	Yes	13	16	
Treatment history (n)	No	98	99	0.996
	Yes	110	111	
*Accompanying syndromes*				
Phlegm-complicated Syndrome (n)	No	148	144	0.565
	Yes	60	66	
Food-stagnation-complicated Syndrome (n)	No	74	69	0.558
	Yes	134	141	
Fright-complicated Syndrome (n)	No	167	178	0.228
	Yes	41	32	
Influenza virus *RT-PCR testing results*				
RT-PCR for influenza (n)	Negative	52	62	0.491
	Positive	154	147	
	Not examined	2	1	
RT-PCR for influenza A (n)	Negative	91	96	0.797
	Positive	115	113	
	Not examined	2	1	
RT-PCR for influenza B (n)	Negative	167	174	0.673
	Positive	39	35	
	Not examined	2	1	

### 3.3 Primary endpoint

**FIGURE 2:**
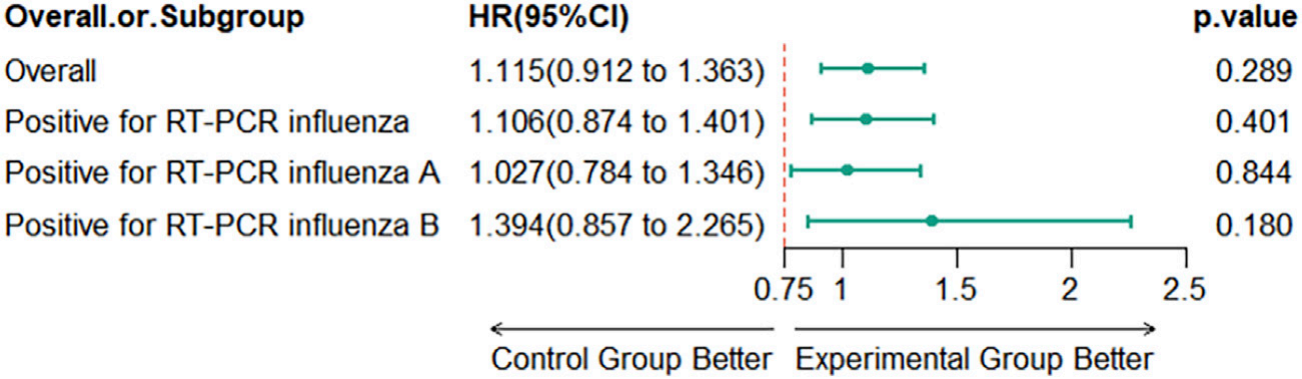
Non-inferiority Test Results for the Overall Population and Subgroups. Non-inferiority margin Δ = 0.75.

**FIGURE 3:**
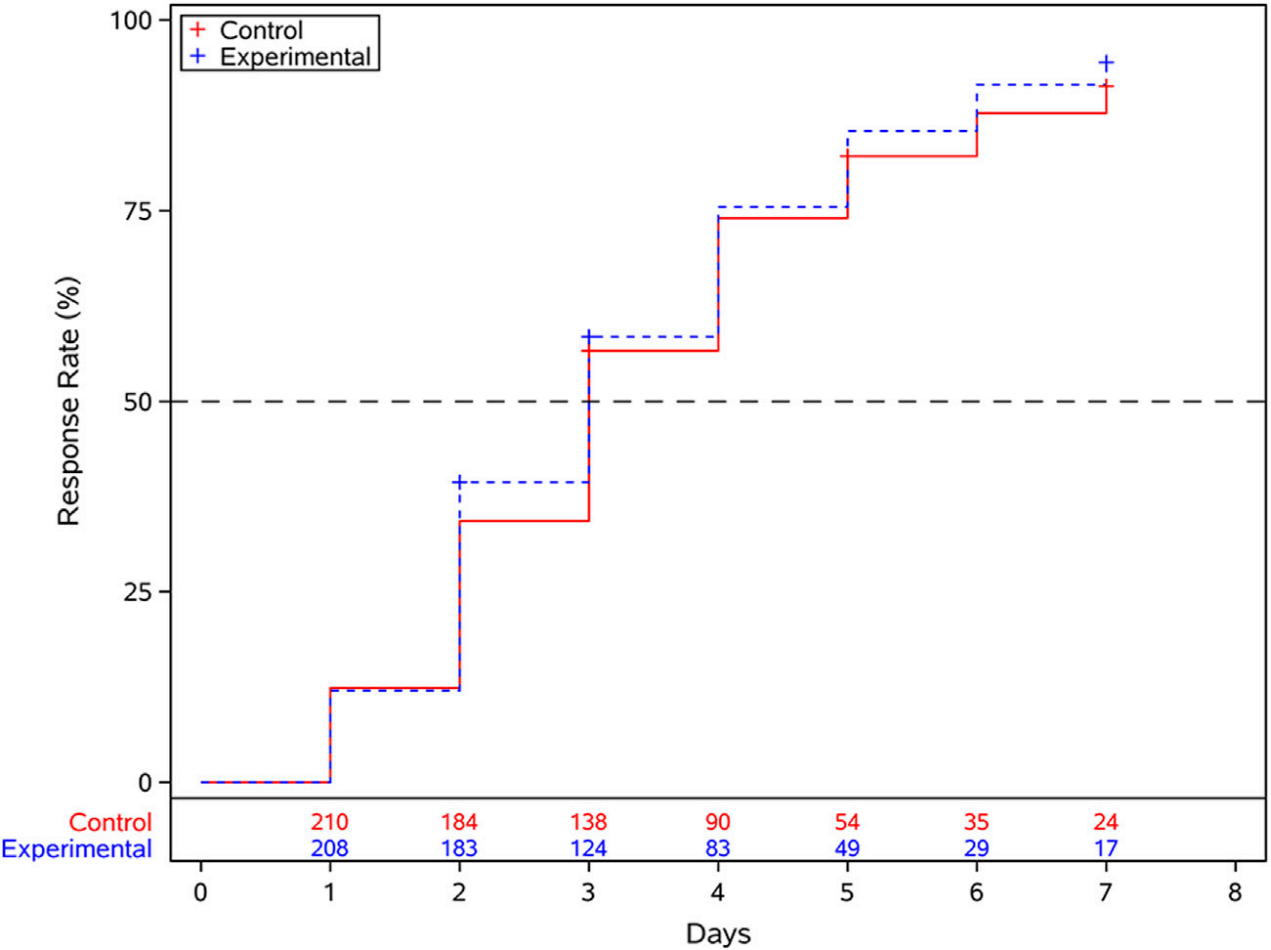
Kaplan-Meier curves for time to clinical recovery.

The Cox proportional hazards regression model, adjusted for disease course effects, indicated an HR of 1.115 (95% CI: 0.912–1.363) for the experimental group compared with the control group. As the lower bound of the 95% CI for the HR (0.912) exceeded the predefined non-inferiority margin (Δ = 0.75), the non-inferiority hypothesis was supported, demonstrating that the efficacy of the experimental group was not inferior to that of the control group. Subgroup analyses revealed that among participants who tested positive for RT-PCR influenza, RT-PCR influenza A, or RT-PCR influenza B, the results were consistent with those observed in the overall population (Figure 2). Additionally, supplementary analyses of potential subgroup interactions revealed no statistically significant interaction effects. (Supplementary Material S5) Kaplan-Meier survival analysis demonstrated that the median time to clinical recovery was 3 days in both the treatment and control groups across the overall population (Figure 3).

### 3.4 Secondary endpoints

**FIGURE 4:**
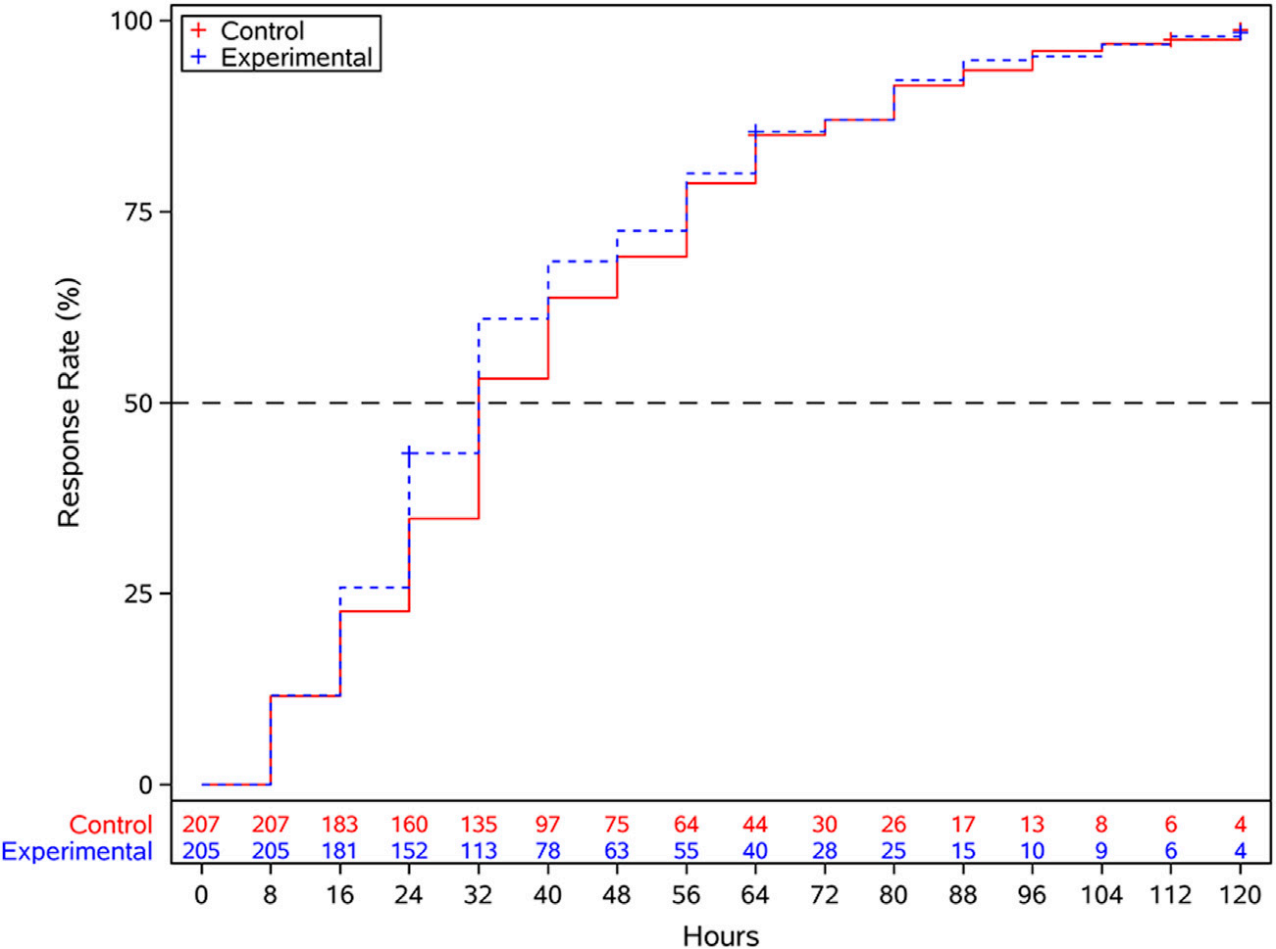
Kaplan-Meier curves for time to complete fever resolution.

The median time to complete fever resolution was 32 h (16–56 h) in the experimental group and 32 h (24–56 h) in the control group. Log-rank test indicated no statistically significant difference between the groups (*P* = 0.407) (Figure 4). The AUC for CARIFS symptom dimension scores over time showed no statistically significant difference between the two groups (*P* = 0.211). No significant differences were observed between the groups in the efficacy rates of TCM syndromes of Wind-Heat Invading the Defense Syndrome (*P* = 0.076) and Fright-complicated Syndrome (*P* = 0.168); however, significant differences were found in Phlegm-complicated Syndrome (*P* = 0.008) and Food-stagnation-complicated Syndrome (*P* = 0.024). The disappearance rates for individual symptoms, such as red and swollen pharynx, cough, copious sputum or audible phlegm sounds in the throat, and lack of appetite, showed statistically significant differences between the groups (*P* < 0.05), while no significant differences were observed for other symptoms. No statistically significant differences were observed between the experimental and control groups in the incidence of complications (*P* = 0.346) and severe and critical influenza (*P* = 0.622). The usage rate (*P* = 0.17), total usage amount (*P* = 0.452), and usage times (*P* = 0.375) of acetaminophen oral suspension showed no statistically significant differences between the two groups. There were no statistically significant differences in viral negative conversion rate between groups for either influenza A (*P* = 0.767) or influenza B (*P* = 0.817) at post-treatment (5 ± 1 day after medication) (Table 2).

**TABLE 2:** Comparison of secondary endpoints between the two groups.

Secondary endpoints	Description	Experimental group (n = 208)	Control group (n = 210)	*p-value*
*Complete fever resolution time (h)*	Median (Q1-Q3)	32 h (16–56 h)	32 h (24–56 h)	0.407
*TCM syndrome efficacy*				
Wind-Heat Invading the Defense Syndrome (n)	Effective	193	184	0.076
	Ineffective	15	26	
Phlegm-complicated Syndrome (n)	Effective	57	52	0.008
	Ineffective	3	14	
Food-stagnation-complicated Syndrome (n)	Effective	124	118	0.024
	Ineffective	10	23	
Fright-complicated Syndrome (n)	Effective	38	26	0.168
	Ineffective	3	6	
*Disappearance rates for individual symptoms*				
Fever (n)	Disappear	195	197	0.980
	Persisted	13	13	
Headache (n)	Disappear	99	106	0.717
	Persisted	10	9	
Nasal congestion (n)	Disappear	68	68	0.292
	Persisted	26	36	
Rhinorrhea (n)	Disappear	99	72	0.070
	Persisted	43	50	
Sneezing (n)	Disappear	33	36	0.679
	Persisted	2	4	
Red and swollen pharynx (n)	Disappear	127	110	0.040
	Persisted	45	63	
Cough (n)	Disappear	98	74	0.004
	Persisted	80	112	
Copious sputum or audible phlegm sounds in the throat (n)	Disappear	50	43	0.020
	Persisted	10	23	
Distension and fullness in the epigastrium and abdomen (n)	Disappear	134	139	0.499
	Persisted	0	2	
Lack of appetite (n)	Disappear	116	101	0.002
	Persisted	18	40	
Foul breath (n)	Disappear	130	136	1.000
	Persisted	4	5	
Nausea and vomiting (n)	Disappear	131	141	0.114
	Persisted	3	0	
Acidic and putrid vomitus (n)	Disappear	134	141	-
	Persisted	0	0	
Acidic and malodorous stools (n)	Disappear	132	141	0.237
	Persisted	2	0	
Abdominal pain (n)	Disappear	133	141	0.487
	Persisted	1	0	
Diarrhea (n)	Disappear	132	141	0.237
	Persisted	2	0	
Constipation (n)	Disappear	133	138	0.623
	Persisted	1	3	
Fright and shrieking (n)	Disappear	41	32	-
	Persisted	0	0	
Restlessness and agitation (n)	Disappear	34	25	0.605
	Persisted	7	7	
*Incidences of complications and severe and critical influenza*				
Incidences of complications (n)	No	201	199	0.346
	Yes	7	11	
Incidences of severe and critical influenza (n)	No	206	209	0.622
	Yes	2	1	
*The usage of acetaminophen*				
Usage rate	No	64	52	0.170
	Yes	144	158	
Total usage amount		14.649 ± 17.114	15.882 ± 16.148	0.452
Usage times		1.941 ± 1.944	2.116 ± 2.045	0.375
*Viral negative conversion rate*				
RT-PCR for influenza A (n = 228)	Positive-Negative	80	80	0.767
	Positive-Positive	25	26	
	Positive-Not examined	10	7	
RT-PCR for influenza B (n = 74)	Positive-Negative	25	25	0.817
	Positive-Positive	10	7	
	Positive-Not examined	4	3	

### 3.5 Safety

This study reported 43 AE (21 in the experimental group and 22 in the control group), primarily consisting of respiratory infections (e.g., bronchitis) and abnormalities in laboratory parameters (e.g., aspartate aminotransferase levels). Seven ADR were documented, with two cases occurring in the experimental group and five in the control group. Additionally, six serious adverse events (SAE) were identified, comprising two cases in the experimental group and four in the control group, all classified as mild in severity. The incidence rates of AE (*P* = 0.885), ADR (*P* = 0.685), and SAE (*P* = 0.449) did not differ significantly between the groups (Table 3).

**TABLE 3:** Comparison of safety outcomes between the two groups.

Observation indicators	Description	Experimental group (n = 209)	Control group (n = 210)	p-value
Adverse events	No	188 (89.95%)	188 (89.52%)	0.885
	Yes	21 (10.05%)	22 (10.48%)	
Serious adverse events	No	207 (99.04%)	206 (98.10%)	0.685
	Yes	2 (0.96%)	4 (1.90%)	
Adverse drug reactions	No	207 (99.04%)	205 (97.62%)	0.449
	Yes	2 (0.96%)	5 (2.38%)	

## 4 Discussion

This nationwide multicenter clinical trial demonstrated that XFQOL is non-inferior to Oseltamivir in terms of clinical recovery time for the treatment of pediatric influenza (Wind-Heat Invading the Defense Syndrome). The efficacies of XFQOL in terms of complete fever resolution time, TCM syndrome efficacy (Wind-Heat Invading the Defense and Fright-complicated Syndrome), disappearance rates for individual symptoms, incidence of complications and severe and critical cases, the usage of acetaminophen, and viral negative conversion rate were all no statistically significant differences to those of Oseltamivir. These results suggest that the efficacy of XFQOL in these aspects is comparable to that of Oseltamivir. Notably, the experimental group demonstrated superior efficacy in treating TCM syndromes of Phlegm-complicated and Food-stagnation-complicated, with statistically significant differences compared to the control group. This suggests a trend that XFQOL may be more effective than Oseltamivir in treating these two TCM patterns. The incidence of AE and ADR observed in both groups was low, with safety profiles within acceptable limits.

Against the backdrop of high pediatric influenza incidence, continuous viral mutation, and increasing resistance, this study conducts XFQOL primarily due to its numerous advantages as a therapeutic agent. The core concept of pediatric TCM, “insufficient pulmonary and splenic functions but surplus cardiac and hepatic activity,” originated with Qian Yi during the Song dynasty and was further refined by Wan Quan in the Ming dynasty, serving as the theoretical basis for this formulation. TCM posits that pediatric pulmonary and splenic functions are typically weak, often leading to symptoms like coughing, sputum production, abdominal distension, vomiting, and diarrhea when children affected by common cold or influenza. In contrast, the cardiac and hepatic functions tend to be hyperactive, predisposing children to symptoms such as fright, shrieking, irritability, and restlessness. Modern medicine has documented comparable observations concerning these clinical manifestations. Most pediatric influenza cases without complications resolve within 3–7 days, although cough symptoms often persist for 1–2 weeks (Wang X. et al., 2024). Infants and preschool-aged children frequently present with gastrointestinal symptoms such as abdominal pain, poor appetite, and diarrhea, which are particularly prominent in some cases of influenza B infection (Hu, 2021; Wang et al., 2022). Febrile seizures are among the most common neurological complications of influenza. In Japan and other Asian countries, influenza A virus is a leading cause of febrile seizures in children (Millichap and Millichap, 2004). However, biomedicine lacks specific treatment options for these accompanied symptoms. This medication fully leverages the advantages of TCM, including its multi-component, multi-target, and multi-pathway properties (Zhang et al., 2024). The formulation enhances clearing heat, removing toxin and reducing fever effects, while incorporating drugs targeting common accompanied symptoms based on the unique constitution and pathological characteristics of children. This unique design is not found in similar drugs, thereby expanding the clinical application scope of XFQOL.

Preliminary pharmacological studies suggest that XFQOL exhibits a certain degree of efficacy in the treatment of influenza. Research demonstrates that this medication reduces viral load, lung index, and serum IL-6 levels in mice with influenza-induced pneumonia, and effectively ameliorates the pathological damage to lung tissues caused by the virus (Sun et al., 2022). *Yinqiao Powder*, the cornerstone prescription of XFQOL, is derived from the classic Chinese medical text “Differentiation of Patterns in Warm Febrile Diseases” and has been consistently recommended in successive editions of TCM guidelines for pediatric influenza (Ma R. et al., 2024). Studies have demonstrated that *Yinqiao Powder* exerts antiviral effects against influenza and mitigates inflammation by modulating the TLR7/NF-κB signaling pathway (Fu et al., 2018). *Gypsum Fibrosum* and *Radix Bupleuri* are pivotal antipyretic agents in pediatric TCM with over a millennium of medicinal heritage, their efficacy and safety widely recognized and validated through both historical use and modern research in China. *Gypsum Fibrosum* lowers body temperature and alleviates fever symptoms by modulating temperature-regulating mediators (IL-6, IL-1β, TNF-α, and PGE2), the NF-κB signaling pathway (Ye et al., 2020). *Radix Bupleuri* extract and saikosaponins demonstrate antiviral, anti-inflammatory, and immunomodulatory effects through the NF-κB, MAPK, or other pathways (Yuan et al., 2017). *Radix Isatidis*, *Radix Scutellariae*, *Fructus Gardeniae*, and *Radix Paeoniae Rubra* possess heat-clearing and detoxifying properties and are widely used for treating colds, influenza, and fever. For example, *Radix Scutellariae* contains hundreds of active metabolites, predominantly flavonoids, exhibiting extensive pharmacological activities, including antiviral and anti-inflammatory effects (Ma W. et al., 2024; Song et al., 2020). The primary component of *Semen Armeniacae Amarum*, amygdalin, exhibits substantial pharmacological activity and is commonly used to relieve cough and reduce phlegm (Zhang et al., 2022). *Fructus Aurantii* and *Massa Medicata Fermentata* are commonly used as a medicinal pair for promoting digestion and alleviating food stagnation. *Fructus Aurantii* contains over 60 identified metabolites, including flavonoids, alkaloids, and coumarins, and is widely applied in managing indigestion, loss of appetite, and bloating (Gao et al., 2020). The bioactive metabolites of Massa Medicata Fermentata are concentrated in the n-butanol-soluble fraction, which contains metabolites that enhance intestinal smooth muscle contraction and accelerate peristalsis, thus improving digestive function (Zhang et al., 2021). *Bombyx Batryticatus* and *Radix Saposhnikoviae* are representative TCM known for their anticonvulsant effects. *Bombyx Batryticatus* contains various bioactive metabolites, including flavonoids, ammonium oxalate, and beauvericin, all of which are associated with anticonvulsant properties (Xing et al., 2019). *Radix Saposhnikoviae* exhibits multiple pharmacological activities, including anticonvulsant, sedative, antipyretic, and analgesic effects (Chen et al., 2021).

The inclusion criteria in our study were based on the diagnostic standards specified in the 2020 edition of the *Guidelines for diagnosis and treatment of influenza*. Children presenting with typical influenza-like symptoms and testing positive on rapid antigen diagnostic testing (RADT) were deemed eligible for enrollment and randomized to treatment (General Office of the National Health Commission and Office of the National Administration of Traditional Chinese Medicine, 2021). Specimens for RT-PCR were collected simultaneously with RADT to enable subsequent subgroup analyses. In this study, the concordance between RADT and RT-PCR was approximately 72.5%, which may be attributed to the sensitivity and specificity of the RADT, as well as the underlying prevalence of influenza. Previous studies have suggested that when influenza prevalence is ≥ 15%, the positive predictive value of RADT approaches 80%, but drops to below 70% when prevalence falls below 10% (Grijalva et al., 2007; Mutnal et al., 2021). Another study reported a 71.4% agreement (3,300/4,622) between influenza A RADT and real-time reverse transcription polymerase chain reaction assays (Zhou et al., 2021). We conducted a subgroup analysis of the primary endpoint, and the results showed that each subgroup yielded conclusions consistent with those of the overall population.

The human experience data of TCM are important evidence to support the evaluation of new TCM drugs. Consequently, TCM clinical trials primarily focus on efficacy, in contrast to the emphasis on safety observed in studies of synthetic drugs (National Medical Products Administration, 2022). The study protocol includes eight evaluation indicators, both subjective and objective, in order to provide the most comprehensive assessment of the efficacy of the trial drug. The objective of this study was to evaluate the efficacy of XFQOL in shortening the duration of illness and fever, as well as in improving clinical symptoms (Hu, 2021). Therefore, viral negative conversion rate was not included as the primary evaluation indicator. This study provides the first comprehensive and systematic evaluation of accompanied symptoms in pediatric influenza, filling a gap in the existing research in this area. Under the double-blind design of the multi-center layout, the results of this study demonstrate high external validity and reliability, providing high-quality evidence for the large-scale clinical application of the drug and highlight the translational value of traditional medicine within the modern medical system. However, several limitations remain: First, recovery from symptoms such as coughing often requires 1–2 weeks. The treatment course and follow-up period in this study were limited to 7 days, potentially hindering the observation of final symptom resolution. Second, the study did not include a placebo control, which limits the ability to evaluate the absolute efficacy of the investigational drug. Future studies should further evaluate this drug through multicenter randomized controlled trials or real-world studies across various clinical settings (including outpatient and inpatient care), to systematically compare its efficacy with established standard therapies or analogous traditional Chinese medicines, while comprehensively assessing its effectiveness and safety profiles in diverse healthcare environments.

In conclusion, the efficacy of XFQOL in treating pediatric influenza (Wind-Heat Invading the Defense Syndrome) is non-inferior to Oseltamivir with respect to clinical recovery time. Additionally, its effectiveness in terms of fever reduction, symptom alleviation, incidences of complications and severe and critical influenza, the usage of acetaminophen, and viral negative conversion rate is comparable to that of Oseltamivir. Furthermore, it demonstrates good safety, suggesting its potential for clinical application.

## Data Availability

The original contributions presented in the study are included in the article/Supplementary Material, further inquiries can be directed to the corresponding authors.
