# Supplementary material for: Efficacy and safety of Xiao’er Fengre Qing oral liquid versus Oseltamivir in treating pediatric influenza (wind-heat invading the defense syndrome): a multicenter, randomized, non-inferiority trial
Source: Front Pharmacol. 2025 May 22;16:1584003. doi: 10.3389/fphar.2025.1584003 (PMC12137347; doi:10.3389/fphar.2025.1584003)
Supplement: Supplementary file 1 [file Supplementaryfile1.docx]

Supplementary Material

Supporting information for

**Efficacy and Safety of Xiao'er Fengre Qing Oral Liquid versus Oseltamivir in Treating Pediatric Influenza (Wind-Heat Invading the Defense Syndrome): A Multicenter, Randomized, Non-Inferiority Trial**

# Supplementary information

## Diagnostic criteria

### Diagnostic criteria for influenza

The diagnostic criteria of were formulated based on the *Guidelines for diagnosis and treatment of influenza* (2020), with an emphasis on clinical manifestations, epidemiological history, and etiological examination. Clinical manifestations of influenza are characterized primarily by fever, headache, myalgia, and generalized discomfort. The body temperature can reach 39-40°C, and patients may experience chills and shivering. Common systemic symptoms include muscle and joint pain, fatigue, and loss of appetite. There are often sore throats and dry coughs. Additionally, symptoms such as nasal congestion, rhinorrhea, retrosternal discomfort, facial flushing, and conjunctival injection may also occur. Some patients may present with mild symptoms or be asymptomatic.

1. Clinically diagnosed cases: A clinically diagnosed case is characterized by an epidemiological history (e.g., close contact within 7 days prior to symptom onset with a suspected or confirmed influenza patient, or being one of the cases with clustered onset of influenza-like illness, or having clear evidence of infecting others) and the above clinical manifestations of influenza, while excluding other diseases that could cause influenza-like symptoms.
2. Confirmed cases: If there are the above clinical manifestations of influenza and one or more of the following etiological test results are positive: 1. Positive for influenza virus nucleic acid detection. 2. Positive for influenza antigen detection. 3. Positive for influenza virus culture and isolation. 4. A fourfold or greater increase in influenza virus-specific IgG antibody levels between acute and convalescent phase sera.

### Diagnosis criteria of severe and critical influenza cases

1. One of the following situations is considered a severe case: 1. Persistent high fever (>3 days) accompanied by severe cough, purulent sputum, hemoptysis, or chest pain. 2. Tachypnea, dyspnea, or cyanosis of the lips and mucous membranes. 3. Changes in consciousness such as slow response, drowsiness, restlessness or convulsions. 4. Severe vomiting, diarrhea, and dehydration manifestations. 5. Concurrent pneumonia. 6. Significant exacerbation of underlying comorbid conditions. 7. Other clinical conditions requiring hospitalization.
2. One of the following situations is considered a critical case: 1. Respiratory failure. 2. Acute necrotizing encephalopathy. 3. Shock. 4. Multiple organ dysfunction. 5. Other severe clinical conditions requiring intensive monitoring and treatment.
   1. **Criteria for Traditional Chinese Medicine Syndrome Differentiation**

Diagnostic criteria for Wind-Heat Invading the Defense Syndrome, Phlegm-complicated Syndrome, Food-stagnation-complicated Syndrome, and Fright-complicated Syndrome are defined as follows:

1. Wind-Heat Invading the Defense Syndrome: (i) Symptoms: fever, headache, red and swollen pharynx, cough, nasal congestion, rhinorrhea, and sneezing; (ii) Tongue manifestation and pulse manifestation: red tongue with thin yellow coating, floating and rapid pulse. Diagnosis requires the presence of fever and at least two additional symptoms, with tongue and pulse as reference for differentiation.
2. Phlegm-complicated Syndrome: (i) Symptoms: severe cough with copious sputum or audible phlegm sounds in the throat; (ii) Tongue manifestation and pulse manifestation: thick greasy tongue coating, floating/rapid and slippery- pulse. Diagnosis requires the above symptoms and tongue and pulse for differentiation.
3. Food-stagnation-complicated Syndrome: (i) Symptoms: distension and fullness in the epigastrium and abdomen, loss of appetite, foul breath, nausea and vomiting, acidic and putrid vomitus, acidic and malodorous stools, abdominal pain, diarrhea, and constipation; (ii) Tongue manifestation and pulse manifestation: turbid and greasy tongue coating, slippery pulse. Diagnosis requires at least two symptoms, with tongue and pulse as reference for differentiation.
4. Fright-complicated Syndrome: Symptoms: fright and shrieking, restlessness and agitation, even staring with both eyes, limb convulsions, and cyanosis of the lips; (ii) Tongue manifestation and pulse manifestation: red tongue, floating/rapid and string-like pulse. Diagnosis requires at least one symptom, with tongue and pulse as reference for differentiation.
   1. **Placebo Preparation Process**
      1. **Preparation of the placebo of Xiao’er Fengre Qing Oral Liquid**
5. Take 5L of the liquid medicine of Xiao’er Fengre Qing Oral Liquid and dilute it 20 times to 100L.
6. Add 1% compound natural pigment and stir evenly.
7. Fill the solution into containers
8. Sterilization.
9. Packaging.
   - 1. **Preparation of the placebo of Oseltamivir**
10. Crushing: Grind 20kg of white granulated sugar into a powder with a particle size of 80-100 mesh.
11. Making soft material: Add sucrose powder into the trough mixer, measure 50% ethanol at 10% of the proportion of sucrose, and stir for about 3 - 5 minutes until the color is uniform and the dry and wet conditions are appropriate.
12. Granulation: Use a granulator with a 30-mesh screen (0.6mm). Continuously and evenly add materials into the hopper until the materials extrude through the screen mesh in strips and break naturally. Spread the prepared wet granules evenly.
13. Drying: Set the oven temperature at 60°C, and the loss on drying is ≤ 2%.
14. Pellet Shaping: Transfer the dried granules to a rotary granulator with a 20-mesh sieve for shaping.
15. Screening: After pellet shaping, screen the granules using a 24-mesh upper sieve and a 40-mesh lower sieve. Select granules between 24 and 40 mesh.
16. Mixing: Put the mixed granules into a clean and dry container and send them to the intermediate station for storage and waiting for inspection.
17. Packaging: Carry out inner packaging and outer packaging as required.

# Supplementary Tables

**Supplementary Table 1.** Trial Flow Chart.

| **Research stage**  **Item** | **Baseline: -1 day to 0 day** | **Treatment and Follow - up** | |
| --- | --- | --- | --- |
|  |  | **Treatment observation period: 5±1 days after administration of the drug** | **Follow - up period: until 7 days after drug administration** |
| screen cases | × |  |  |
| Sign the informed consent form | × |  |  |
| Influenza Virus Detection in Throat Swab by Colloidal Gold Method | × |  |  |
| Influenza Virus Detection in Throat Swab by RT-PCR Method | × | × |  |
| General information | × |  |  |
| Physical examination | × | × |  |
| Temperature | The guardians of the child patients fill in the log cards and record the body temperature once before the first dose of medicine and every 8 hours after taking the medicine. | | |
| Canadian Acute Respiratory Illness and Flu Scale (CARIFS) | The guardians of the child patients fill in the log cards and record the CARIFs score once every 24 hours. | | |
| Traditional Chinese Medicine syndrome score | × | × |  |
| Blood and urine routine | × | × | ×* |
| C-reactive protein | × |  |  |
| Electrocardiogram | × | × | ×* |
| Hepatic and renal function | × | × | ×* |
| Myocardial enzyme | ×^#^ | ×^#^ | ×* |
| Distribution and collection of the subjects' log cards | × |  | × |
| Distribution and collection of the trial drugs | × | × |  |
| Record of concomitant medications | × | × | × |
| Adverse event |  | × | ×* |
| Trial Summary |  | × | × |

Note: ×^#^, Optional according to the actual diagnosis and treatment; ×* Follow - up until it returns to normal or the baseline level.

**Supplementary Table 2.** Comparison of Baseline Traditional Chinese Medicine Symptom Scores Between Groups.

| **Traditional Chinese Medicine Symptom** | **Description** | **Experimental group** | **Control group** | ***p-value*** |
| --- | --- | --- | --- | --- |
| Wind-Heat Invading the Defense Syndrome | Mean (SD) | 11.510±3.330 | 11.662±3.679 | 0.658 |
| Phlegm-complicated Syndrome | Mean (SD) | 11.550±2.819 | 11.652±3.635 | 0.709 |
| Food-stagnation-complicated Syndrome | Mean (SD) | 11.970±3.085 | 12.163±3.409 | 0.624 |
| Fright-complicated Syndrome | Mean (SD) | 12.805±3.018 | 14.188±3.847 | 0.149 |

**Supplementary Table 3.** Comparison of Baseline Individual Symptom Scores Between Groups.

| **Symptom** | **Symptom scores** | **Experimental group** | **Control group** | ***p-value*** |
| --- | --- | --- | --- | --- |
| Fever (n) | 2 | 2(0.96%) | 2(0.95%) | 0.711 |
|  | 4 | 124(59.62%) | 129(61.43%) |  |
|  | 6 | 82(39.42%) | 79(37.62%) |  |
| Headache (n) | 0 | 99(47.60%) | 95(45.24%) | 0.648 |
|  | 1 | 57(27.40%) | 60(28.57%) |  |
|  | 2 | 38(18.27%) | 40(19.05%) |  |
|  | 3 | 14(6.73%) | 15(7.14%) |  |
| Nasal congestion (n) | 0 | 114(54.81%) | 106(50.48%) | 0.470 |
|  | 1 | 50(24.04%) | 58(27.62%) |  |
|  | 2 | 38(18.27%) | 40(19.05%) |  |
|  | 3 | 6(2.88%) | 6(2.86%) |  |
| Rhinorrhea (n) | 0 | 66(31.73%) | 88(41.90%) | 0.129 |
|  | 1 | 79(37.98%) | 63(30.00%) |  |
|  | 2 | 58(27.88%) | 51(24.29%) |  |
|  | 3 | 5(2.40%) | 8(3.81%) |  |
| Sneezing (n) | 0 | 173(83.17%) | 170(80.95%) | 0.584 |
|  | 1 | 22(10.58%) | 27(12.86%) |  |
|  | 2 | 13(6.25%) | 13(6.19%) |  |
| Red and swollen pharynx (n) | 0 | 36(17.31%) | 37(17.62%) | 0.970 |
|  | 1 | 103(49.52%) | 102(48.57%) |  |
|  | 2 | 59(28.37%) | 64(30.48%) |  |
|  | 3 | 10(4.81%) | 7(3.33%) |  |
| Cough (n) | 0 | 30(14.42%) | 24(11.43%) | 0.091 |
|  | 1 | 90(43.27%) | 83(39.52%) |  |
|  | 2 | 72(34.62%) | 77(36.67%) |  |
|  | 3 | 16(7.69%) | 26(12.38%) |  |
| Copious sputum or audible phlegm sounds in the throat (n) | 0 | 148(71.15%) | 144(68.57%) | 0.565 |
|  | 1 | 60(28.85%) | 66(31.43%) |  |
| Distension and fullness in the epigastrium and abdomen (n) | 0 | 204(98.08%) | 201(95.71%) | 0.164 |
|  | 1 | 4(1.92%) | 9(4.29%) |  |
| Lack of appetite (n) | 0 | 108(51.92%) | 94(44.76%) | 0.143 |
|  | 1 | 100(48.08%) | 116(55.24%) |  |
| Foul breath (n) | 0 | 190(91.35%) | 192(91.43%) | 0.976 |
|  | 1 | 18(8.65%) | 18(8.57%) |  |
| Nausea and vomiting (n) | 0 | 180(86.54%) | 176(83.81%) | 0.433 |
|  | 1 | 28(13.46%) | 34(16.19%) |  |
| Acidic and putrid vomitus (n) | 0 | 207(99.52%) | 208(99.05%) | 1.000 |
|  | 1 | 1(0.48%) | 2(0.95%) |  |
| Acidic and malodorous stools (n) | 0 | 205(98.56%) | 207(98.57%) | 1.000 |
|  | 1 | 3(1.44%) | 3(1.43%) |  |
| Abdominal pain (n) | 0 | 200(96.15%) | 202(96.19%) | 0.984 |
|  | 1 | 8(3.85%) | 8(3.81%) |  |
| Diarrhea (n) | 0 | 199(95.67%) | 207(98.57%) | 0.076 |
|  | 1 | 9(4.33%) | 3(1.43%) |  |
| Constipation (n) | 0 | 189(90.87%) | 196(93.33%) | 0.350 |
|  | 1 | 19(9.13%) | 14(6.67%) |  |
| Fright and shrieking (n) | 0 | 207(99.52%) | 209(99.52%) | 1.000 |
|  | 1 | 1(0.48%) | 1(0.48%) |  |
| Restlessness and agitation (n) | 0 | 167(80.29%) | 178(84.76%) | 0.228 |
|  | 1 | 41(19.71%) | 32(15.24%) |  |
