# Supplementary material for: Efficacy and safety of Xiao’er Fengre Qing oral liquid versus Oseltamivir in treating pediatric influenza (wind-heat invading the defense syndrome): a multicenter, randomized, non-inferiority trial
Source: Front Pharmacol. 2025 May 22;16:1584003. doi: 10.3389/fphar.2025.1584003 (PMC12137347; doi:10.3389/fphar.2025.1584003)
Supplement: Supplementary file 2 [file Supplementaryfile5.docx]

**Analysis of Subgroup Interactions**

We performed the following supplementary analyses to further investigate the primary endpoint:

1. Subgroup analyses including positive for RT-PCR influenza, positive for RT-PCR influenza A, and positive for RT-PCR influenza B were performed (Table 1);
2. In the RT-PCR-confirmed influenza cases, one co-infected individual (positive for both influenza A and B) was excluded from the analysis (Table 2);
3. Additional covariates including disease duration, sex, and BMI were incorporated in the analysis (Table 3);
4. Restricted mean survival time (RMST) analysis was applied to assess outcome differences across subpopulations, accounting for multiple covariates (Table 4);
5. Three analytical approaches were employed to test the proportional hazards assumption (Table 5).

**Table 1. Supplemental Analysis of Time to Clinical Recovery**

|  | Experimental group | | Control group | | Crude | | P for interaction | Adjusted | | P for interaction |
| --- | --- | --- | --- | --- | --- | --- | --- | --- | --- | --- |
| Subroup | Total | Events(%) | Total | Events(%) | HR(95%CI) | P-value |  | HR(95%CI) | P-value |  |
| Influenza |  |  |  |  |  |  | 0.2511 |  |  | 0.9282 |
| Positive | 154 | 145(94.16%) | 147 | 132(89.79%) | 1.099(0.868～1.391) | 0.4342 |  | 1.108(0.875～1.403) | 0.6821 |  |
| Negative | 52 | 46(88.46%) | 62 | 57(91.94%) | 1.050(0.711～1.551) | 0.8059 |  | 1.085(0.734～1.604) | 0.3940 |  |
| Influenza A |  |  |  |  |  |  | 0.4817 |  |  | 0.4689 |
| Positive | 115 | 108(93.91%) | 113 | 103(91.15%) | 1.026(0.783～1.344) | 0.8512 |  | 1.032(0.788～1.352) | 0.2414 |  |
| Negative | 91 | 83(91.21%) | 96 | 86(89.58%) | 1.150(0.850～1.555) | 0.3654 |  | 1.199(0.885～1.624) | 0.8200 |  |
| Influenza B |  |  |  |  |  |  | 0.6707 |  |  | 0.2956 |
| Positive | 39 | 37(94.87%) | 35 | 30(85.71%) | 1.355(0.835～2.197) | 0.2185 |  | 1.404(0.867～2.275) | 0.6181 |  |
| Negative | 167 | 154(92.22%) | 174 | 159(91.38%) | 1.039(0.832～1.297) | 0.7365 |  | 1.058(0.847～1.322) | 0.1675 |  |

Three cases without RT-PCR test results were excluded from analysis; Adjusted: adjusted for disease duration, subgroups, and group*subgroup.

**Table 2. Supplementary Analysis of Time to Clinical Recovery Among RT-PCR–Confirmed Cases**

|  | Experimental group | | Control group | | Crude | | P for interaction | Adjusted | | P for interaction |
| --- | --- | --- | --- | --- | --- | --- | --- | --- | --- | --- |
| Subroup | Total | Events(%) | Total | Events(%) | HR(95%CI) | P-value |  | HR(95%CI) | P-value |  |
| Influenza |  |  |  |  |  |  | 0.8854 |  |  | 0.2840 |
| Influenza A | 115 | 108(93.91%) | 112 | 102(91.07%) | 1.017(0.776～1.334) | 0.9011 |  | 1.021(0.779～1.338) | 0.1909 |  |
| Influenza B | 39 | 37(94.87%) | 34 | 29(85.29%) | 1.333(0.818～2.173) | 0.2491 |  | 1.384(0.850～2.253) | 0.8811 |  |

One case co-infected with both influenza A and B (RT-PCR positive) was excluded from the analysis; Adjusted: adjusted for disease duration, subgroups, and group*subgroup.

**Table 3. Supplementary Analysis of Time to Clinical Recovery with Adjustment for Additional Covariates**

|  | Estimate | Standard Error | Chisq | P-value | HR(95%CI) |
| --- | --- | --- | --- | --- | --- |
| Model 1 | 0.10837 | 0.10267 | 1.1141 | 0.2912 | 1.114(0.911～1.363) |
| Model 2 | 0.10273 | 0.10273 | 0.9999 | 0.3173 | 1.108(0.906～1.355) |
| Model 3 | 0.10248 | 0.10275 | 0.9949 | 0.3186 | 1.108(0.906～1.355) |

Model 1 was adjusted for disease duration and sex; Model 2 was adjusted for disease duration and body mass index (BMI); Model 3 was adjusted for disease duration, sex, and BMI.

**Table 4. Supplementary Analysis of Time to Clinical Recovery Using the RMST Approach**

|  | Estimate | Standard Error | z | P-value | RMST ratio(95%CI) |
| --- | --- | --- | --- | --- | --- |
| Model 1 | -0.0546 | 0.0510 | -1.0702 | 0.2854 | 0.9468(0.8567～1.0465) |
| Model 2 | -0.0547 | 0.0510 | -1.0707 | 0.2843 | 0.9468(0.8567～1.0464) |
| Model 3 | -0.0545 | 0.0509 | -1.0693 | 0.2849 | 0.9470(0.8570～1.0464) |
| Model 4 | -0.0544 | 0.0509 | -1.0691 | 0.2850 | 0.9470(0.8570～1.0464) |
| Model 5 | -0.0408 | 0.0619 | -0.6586 | 0.5101 | 0.9600(0.8502～1.0839) |

Model 1 was adjusted for disease duration; Model 2 was adjusted for disease duration and sex; Model 3 was adjusted for disease duration and BMI; Model 4 for disease duration, sex, and BMI; Model 5 included only RT-PCR–positive participants and was adjusted for disease duration..

**Table 5. Assessment of the Proportional Hazards Assumption**

| Method | Chisq | P-value |
| --- | --- | --- |
| ZPH t | 0.0627 | 0.8023 |
| ZPH log(t) | 0.0227 | 0.8804 |
| ZPH rank(t) | 0.0115 | 0.9146 |

ZPH, based on the weighted Schoenfeld residuals for checking the proportional hazards assumption.
