# Supplementary material for: Efficacy and safety of Xiao’er Fengre Qing oral liquid versus Oseltamivir in treating pediatric influenza (wind-heat invading the defense syndrome): a multicenter, randomized, non-inferiority trial
Source: Front Pharmacol. 2025 May 22;16:1584003. doi: 10.3389/fphar.2025.1584003 (PMC12137347; doi:10.3389/fphar.2025.1584003)
Supplement: Supplementary file 3 [file Supplementaryfile2.pdf]

Characteristic chromatogram of Xiao’er Fengre Qing Oral Liquid

(Supplementary Material 2)

Contents

1. Experimental Overview 1
2. Experimental Materials 1
2.1 Equipment 1
2.2 Reagents 1
2.3 Reference Substances 1
3. Experimental Methods 1
3.1 Preparation of Test Solution 1
3.2 Chromatographic Conditions 2
3.3 Identification of Characteristic Peaks 2
4. Multi-Batch Sample Analysis 3

1. Experimental Overview

To ensure quality control in the extraction process of Xiao'er Fengre Qing Oral Liquid, a characteristic chromatographic method was established to assess quality from multiple indicators.

2. Experimental Materials

2.1 Equipment

Equipment	Manufacturer	Model	Serial Number	Operational Status
High-Performance Liquid Chromatograph (HPLC)	Waters	Acquily Arc	6181B	Functional

2.2 Reagents

Reagent	Purity	Manufacturer	Batch Number
Acetonitrile	HPLC grade	Lionbridge Bohua (Tianjin) Pharmaceutical Chemicals Co., Ltd.	20221011
Phosphoric Acid	Analytical grade	Yantai Far East Fine Chemicals Co., Ltd.	20210330

2.3 Reference Substances

Name	Manufacturer	Batch Number	Purity (%)
Naringin reference standard	National Institutes for Food and Drug Control	110831-201906	91.5%
Neohesperidin reference standard	National Institutes for Food and Drug Control	110753-201817	96.8%
Baicalin reference standard	National Institutes for Food and Drug Control	111825-201803	96.7%
Paeoniflorin reference standard	National Institutes for Food and Drug Control	111825-201803	96.7%
Forsythin reference standard	National Institutes for Food and Drug Control	111825-201803	96.7%
Arctiin reference standard	National Institutes for Food and Drug Control	110819-201812	95.0%
Baicalin reference standard	National Institutes for Food and Drug Control	110715-202122	94.2%
Gardenoside reference standard	National Institutes for Food and Drug Control	110749-201919	97.1%

3. Experimental Methods

3.1 Preparation of Test Solution

Precisely measure 1.00 mL of Xiao'er Fengre Qing Oral Liquid into a 10 mL volumetric flask, dilute to volume with purified water, mix well, and filter through a 0.22 µm microporous membrane.

3.2 Chromatographic Conditions

Separation was achieved using a Thermo Accucore aQ column (100 mm × 2.1 mm, 2.6 μm) packed with octadecylsilyl (C18) bonded silica gel. The mobile phase consisted of:

Mobile phase A: Acetonitrile

Mobile phase B: 0.1% phosphoric acid aqueous solution

A gradient elution program was employed as detailed in Table 1. The column temperature was maintained at 30°C, with detection wavelength set at 230 nm. The injection volume was 2 μL.

TABLE 1 HPLC gradient elution program

Time/min	Mobile phase A/%	Mobile phaseB/%
0	3	97
5	3	97
10	5	95
20	7	93
35	15	85
55	20	80
70	50	50
75	100	0
80	100	0
82	3	97
90	3	97

3.3 Identification of Characteristic Peaks

The 20 characteristic peaks in the Characteristic chromatogram were identified and assigned through comparative analysis with reference standards and herbal extracts.

(1) Comparison with reference standards

Through comparison with seven representative compounds in the formulation—chlorogenic acid, paeoniflorin, naringin, neoeriocitrin, baicalin, forsythin, and arctiin—the identification results are presented in the table and figure below.

TABLE 2 Identification of Characteristic Peaks of Reference Standard

Characteristic peaks (NO.)	Identification of components
2	chlorogenic acid
6	paeoniflorin
10	naringin
13	neoeriocitrin
16	baicalin
17	forsythin

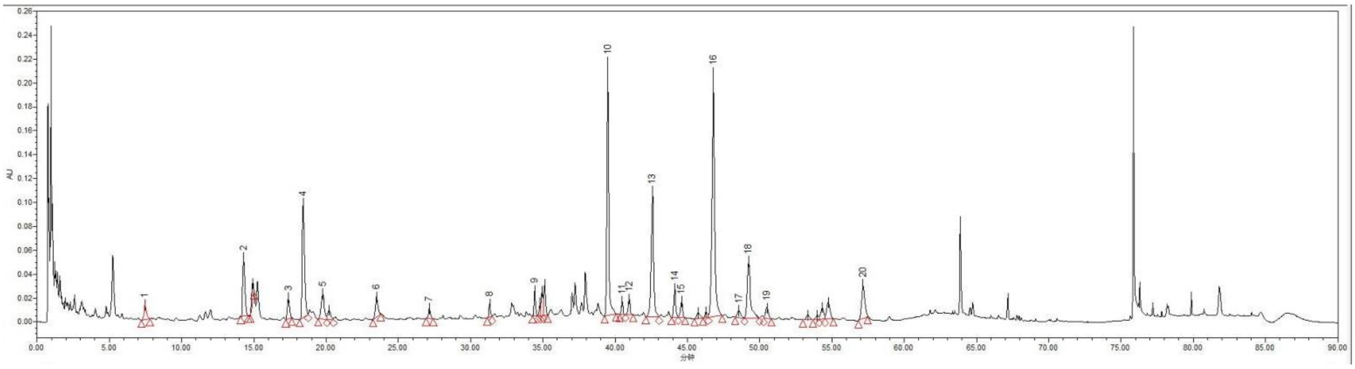

FIGURE 1
Comparison of Characteristic Peaks in the Characteristic Chromatogram of Xiao'er Fengre Qing Oral Liquid. 分钟=Minutes.

(2) Herbal material comparison

The prescription herbs were separately prepared into single-herb reference solutions following the standard preparation method and injected into the high-performance liquid chromatography (HPLC) system for comparative analysis with the reference standard of Xiao’ er Fengre Qing Oral Liquid. After comparison, the characteristic peaks were assigned to corresponding herbal components, as shown in the table below.

TABLE 4 Identification of Characteristic Peaks through Comparison of Medicinal Herbs

Characteristic peaks (NO.)	Corresponding Medicinal Herb	Characteristic peaks (NO.)	Corresponding Medicinal Herb
1	-	11	-
2	Lonicerae Japonicae Flos	12	Scutellariae Radix
3	Gardeniae Fructus	13	Scutellariae Radix
4	Gardeniae Fructus	14	Lonicerae Japonicae Flos
5	Rubiae Radix et Rhizoma	15	Gardeniae Fructus
6	Rubiae Radix et Rhizoma	16	Sophorae Flos
7	Lonicerae Japonicae Flos	17	Nelumbinis Plumula
8	Saposhnikoviae Radix	18	Polygoni Cuspidati Rhizoma et Radix
9	Saposhnikoviae Radix	19	-
10	Scutellariae Radix	20	Sophorae Flos

4. Multi-Batch Sample Analysis

A total of 16 batches of commercially available Xiao’er Fengre Qing Oral Liquid samples were taken, and test solutions were prepared according to the specified preparation method. A 2μL aliquot of each test solution was injected into a high-performance liquid chromatography (HPLC) system for measurement. The results showed that the relative retention time had an RSD ≤0.1738%. The

selected characteristic peaks were stably reproduced across multiple batches of samples, with no significant variation observed between different batches of Xiao'er Fengre Qing Oral Liquid, indicating stable manufacturing processes. (TABLE 4 and FIGURE 2)

TABLE 4 Results of the Inter-batch Difference Test for the Characteristic Chromatogram of Xiao'er Fengre Qing Oral Liquid

Peak number	Inter-batch variation																Average	Standard Deviation（%）
	004	005	006	007	008	009	010	011	012	013	014	015	016	017	018	019		
1	0.1734	0.1734	0.1735	0.1735	0.1730	0.1731	0.1732	0.1732	0.1733	0.1735	0.1736	0.1741	0.1730	0.1730	0.1734	0.1735	0.1734	0.1738
2	0.3344	0.3343	0.3344	0.3343	0.3336	0.3339	0.3337	0.3338	0.3337	0.3338	0.3336	0.3345	0.3337	0.3337	0.3341	0.3343	0.3340	0.0973
3	0.4075	0.4075	0.4075	0.4076	0.4069	0.4073	0.4071	0.4073	0.4072	0.4073	0.4073	0.4084	0.4069	0.4071	0.4073	0.4074	0.4074	0.0823
4	0.4309	0.4308	0.4309	0.4310	0.4306	0.4309	0.4308	0.4308	0.4309	0.4310	0.4310	0.4319	0.4305	0.4305	0.4308	0.4309	0.4309	0.0724
5	0.4602	0.4602	0.4604	0.4604	0.4605	0.4607	0.4607	0.4608	0.4608	0.4610	0.4611	0.4618	0.4604	0.4603	0.4603	0.4603	0.4606	0.0908
6	0.5502	0.5499	0.5502	0.5503	0.5499	0.5502	0.5500	0.5501	0.5501	0.5503	0.5503	0.5509	0.5499	0.5497	0.5501	0.5498	0.5501	0.0495
7	0.6369	0.6367	0.6369	0.6369	0.6368	0.6370	0.6369	0.6371	0.6371	0.6372	0.6373	0.6378	0.6366	0.6365	0.6369	0.6368	0.6370	0.0467
8	0.7370	0.7370	0.7370	0.7370	0.7364	0.7365	0.7365	0.7364	0.7364	0.7364	0.7361	0.7363	0.7365	0.7364	0.7369	0.7369	0.7366	0.0414
9	0.8093	0.8093	0.8093	0.8093	0.8091	0.8092	0.8092	0.8092	0.8092	0.8093	0.8091	0.8093	0.8093	0.8091	0.8093	0.8092	0.8092	0.0085
10	0.9274	0.9275	0.9275	0.9275	0.9274	0.9276	0.9274	0.9275	0.9274	0.9275	0.9274	0.9275	0.9276	0.9274	0.9275	0.9275	0.9275	0.0067
11	0.9508	0.9510	0.9509	0.9509	0.9507	0.9508	0.9507	0.9507	0.9507	0.9508	0.9506	0.9506	0.9509	0.9507	0.9509	0.9509	0.9508	0.0139
12	0.9623	0.9625	0.9624	0.9624	0.9622	0.9623	0.9623	0.9622	0.9622	0.9623	0.9622	0.9622	0.9624	0.9622	0.9624	0.9624	0.9623	0.0092
13（S）	1.0000	1.0000	1.0000	1.0000	1.0000	1.0000	1.0000	1.0000	1.0000	1.0000	1.0000	1.0000	1.0000	1.0000	1.0000	1.0000	1.0000	0.0000
14	1.0358	1.0358	1.0359	1.0357	1.0356	1.0357	1.0357	1.0357	1.0356	1.0357	1.0356	1.0356	1.0356	1.0356	1.0358	1.0358	1.0357	0.0091
15	1.0474	1.0474	1.0475	1.0473	1.0472	1.0472	1.0472	1.0471	1.0471	1.0471	1.0471	1.0471	1.0471	1.0471	1.0474	1.0473	1.0472	0.0121
16	1.0983	1.0982	1.0984	1.0983	1.0980	1.0982	1.0981	1.0980	1.0980	1.0981	1.0981	1.0981	1.0980	1.0980	1.0983	1.0981	1.0981	0.0114
17	1.1391	1.1391	1.1393	1.1392	1.1389	1.1391	1.1391	1.1391	1.1390	1.1391	1.1392	1.1395	1.1390	1.1390	1.1392	1.1390	1.1391	0.0120
18	1.1546	1.1545	1.1547	1.1547	1.1546	1.1548	1.1547	1.1548	1.1547	1.1549	1.1550	1.1555	1.1546	1.1546	1.1548	1.1545	1.1547	0.0201
19	1.1842	1.1841	1.1843	1.1844	1.1844	1.1844	1.1844	1.1845	1.1843	1.1845	1.1847	1.1852	1.1843	1.1842	1.1843	1.1841	1.1844	0.0231
20	1.3399	1.3398	1.3401	1.3401	1.3400	1.3401	1.3402	1.3403	1.3401	1.3403	1.3406	1.3414	1.3401	1.3399	1.3401	1.3400	1.3402	0.0278

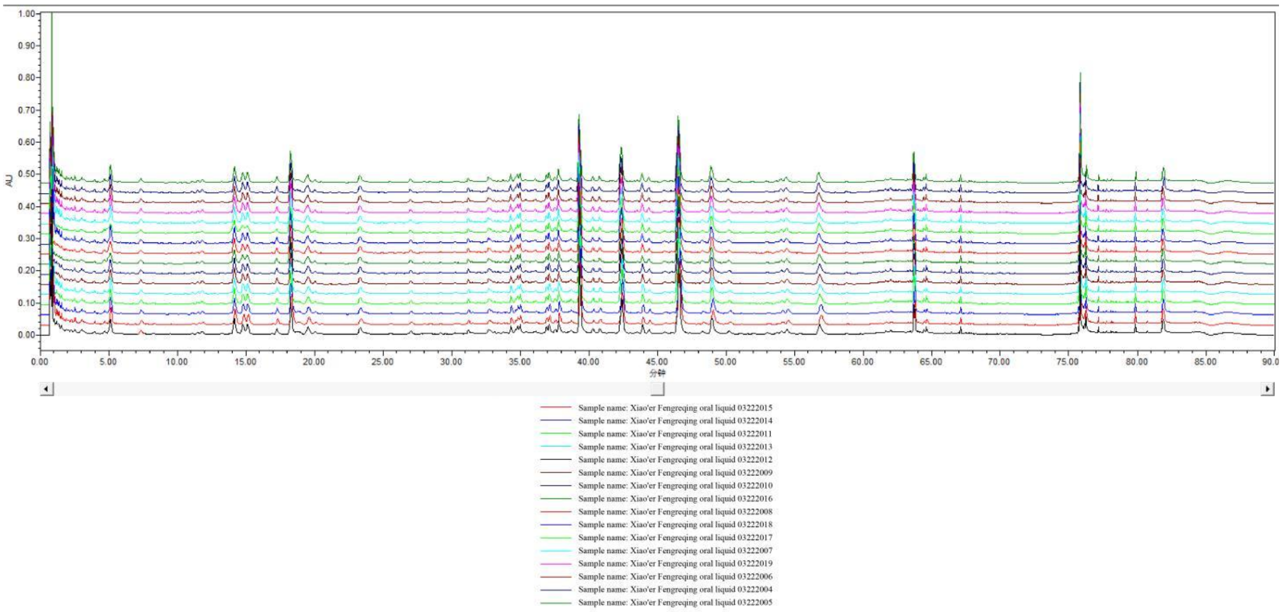

FIGURE 2

Characteristic Chromatograms of 16 Batches of Xiao'er Fengre Qing Oral Liquid.分钟=Minutes.
