# Supplementary material for: Efficacy and safety of Xiao’er Fengre Qing oral liquid versus Oseltamivir in treating pediatric influenza (wind-heat invading the defense syndrome): a multicenter, randomized, non-inferiority trial
Source: Front Pharmacol. 2025 May 22;16:1584003. doi: 10.3389/fphar.2025.1584003 (PMC12137347; doi:10.3389/fphar.2025.1584003)
Supplement: Supplementary file 4 [file Supplementaryfile4.pdf]

2017 Edition 01

Appendix: 01-050-1 File No.: II01-050(2016) D

Handan Pharmaceutical Co., Ltd.

Certificate of Analysis

Report No.:

**Product Name**	Xiao'er Fengreqing Oral Liquid	**Batch No.**	XE23001	**Receipt Date**	2023-08-16
**Package**	Glass vial + Paper box	**Production Site**	Comprehensive Preparation Workshop	**Report Date**	2023-08-23
**Specification**	10 mL × 6 vials × 6 boxes	**Purpose**	Release Testing	**Batch Size**	9,360 vials
**Quantity Tested**	40 vials	**Test Items**	Full Testing	**Submitted By**	Niu Xiaocui

**Testing Standard**	National Medical Products Administration (NMPA) Pharmaceutical Standard YBZ05062017		
**Test Item**	**Specification**	**Result**	**Conclusion**
**Description**	Brown-red to brownish liquid; sweet with slight bitterness	Brown liquid; sweet with slight bitterness	Complies
**Identification**			
	The thin-layer chromatography shall show a spot corresponding to arctiin	Shows a corresponding spot	Complies
	The thin-layer chromatography shall show a spot corresponding to baicalin	Shows a corresponding spot	Complies
	The thin-layer chromatography shall show a spot corresponding to geniposide	Shows a corresponding spot	Complies
	The thin-layer chromatography shall show a spot corresponding to forsythin	Shows a corresponding spot	Complies
**Tests**			
**Relative density**	≥1.07	1.13	Complies
**pH**	4.5-6.0	5.3	Complies
**Fill volume variation**	Shall comply with the requirements	Complies	Complies
**Microbial limits**	Shall comply with the requirements	Complies	Complies
**Assay**			
	Contains not less than 0.70 mg of geniposide per 1 mL	1.78 mg/mL	Complies
**CONCLUSION**	The product complies with the requirements of NMPA Standard YBZ05062017.		
**Remarks**			

Tested by: Reviewed by: Approved by:

Handan Pharmaceutical Co., Ltd.

Certificate of Analysis

Report No.:

**Product Name**	Xiao'er Fengreqing Oral Liquid (Placebo)	**Batch No.**	XE23002	**Receipt Date**	2023-08-17
**Package**	Glass vial + Paper box	**Production Site**	Comprehensive Preparation Workshop	**Report Date**	2023-08-23
**Specification**	10 mL × 6 vials × 6 boxes	**Purpose**	Release Testing	**Batch Size**	9,000 vials
**Quantity Tested**	40 vials	**Test Items**	Full Testing	**Submitted By**	Niu Xiaocui
**Testing Standard**	National Medical Products Administration (NMPA) Pharmaceutical Standard YBZ05062017				

**Test Item**	**Specification**	**Result**	**Conclusion**
**Tests**			
**Fill volume variation**	Shall comply with the requirements	Complies	Complies
**Microbial limits**	Shall comply with the requirements	Complies	Complies

**CONCLUSION**	The product complies with the requirements of NMPA Standard YBZ05062017.
**Remarks**	

Tested by: Reviewed by: Approved by:

Handan Pharmaceutical Co., Ltd.

Certificate of Analysis

Report No.:

**Product Name**	Oseltamivir Phosphate Granules	**Batch No.**	6002305056	**Receipt Date**	2023-05-08
**Package**	Composite film	**Production Site**	Comprehensive Preparation Workshop	**Report Date**	2023-05-14
**Specification**	15 mg/bag	**Purpose**	Release Testing	**Batch Size**	15,000 vials
**Quantity Tested**	30 bags	**Test Items**	Full Testing	**Submitted By**	Niu Xiaocui
**Testing Standard**	*Chinese Pharmacopoeia* 2020 Edition, Volume IV General Chapters				

**Test Item**	**Specification**	**Result**	**Conclusion**
**Tests**			
**Water content**	≤8.0%	0.1%	Complies
**Fill volume variation**	Shall comply with the requirements	Complies	Complies
**Microbial limits**	Shall comply with the requirements	Complies	Complies

**CONCLUSION**	The product complies with the requirements of *Chinese Pharmacopoeia* 2020 Edition, Volume IV General Chapters.
**Remarks**	

Tested by: Reviewed by: Approved by:

Handan Pharmaceutical Co., Ltd.

Certificate of Analysis

Report No.:

**Product Name**	Oseltamivir Phosphate Granules (Placebo)	**Batch No.**	M6002305056	**Receipt Date**	2023-08-18
**Package**	Composite film	**Production Site**	Comprehensive Preparation Workshop	**Report Date**	2023-08-24
**Specification**	15 mg/bag	**Purpose**	Release Testing	**Batch Size**	15,600 vials
**Quantity Tested**	30 bags	**Test Items**	Full Testing	**Submitted By**	Niu Xiaocui
**Testing Standard**	*Chinese Pharmacopoeia* 2020 Edition, Volume IV General Chapters				

**Test Item**	**Specification**	**Result**	**Conclusion**
**Tests**			
**Water content**	≤8.0%	0.04%	Complies
**Fill volume variation**	Shall comply with the requirements	Complies	Complies
**Microbial limits**	Shall comply with the requirements	Complies	Complies
**CONCLUSION**	The product complies with the requirements of *Chinese Pharmacopoeia* 2020 Edition, Volume IV General Chapters.		
**Remarks**			

Tested by: Reviewed by: Approved by:
