# Supplementary material for: Efficacy and safety of Xiao’er Fengre Qing oral liquid versus Oseltamivir in treating pediatric influenza (wind-heat invading the defense syndrome): a multicenter, randomized, non-inferiority trial
Source: Front Pharmacol. 2025 May 22;16:1584003. doi: 10.3389/fphar.2025.1584003 (PMC12137347; doi:10.3389/fphar.2025.1584003)
Supplement: Supplementary file 5 [file Supplementaryfile3.pdf]

2017 版 01

附表：01-050-1　　　　　　　　　　　　　　　　　　　　文件编号：II01-050（2016）D

邯郸制药股份有限公司

产品检验报告书

报告书编号：

检品名称	小儿风热清口服液	批　　号	XE23001	收检日期	2023 年 08 月 16 日
包　　装	玻璃瓶及纸盒	生产车间	综合制剂车间	报告日期	2023 年 08 月 23 日
规　　格	10 毫升×6 支×6 盒	检验目的	出厂检验	产　　量	9360 支
检品数量	40 支	检验项目	全检	送 检 人	牛肖翠
检验依据	国家食品药品监督管理局药品标准 YBZ05062017				

检验项目	标准规定	检验数据	项目结论
[性状]	应为棕红色至棕褐色液体；味甜、微苦	本品为棕褐色液体；味甜、微苦	符合规定
[鉴别]	（1）薄层色谱中应显与牛蒡苷相对应的斑点	显相对应的斑点	符合规定
	（2）薄层色谱中应显与黄芩苷相对应的斑点	显相对应的斑点	符合规定
	（3）薄层色谱中应显与栀子苷相对应的斑点	显相对应的斑点	符合规定
	（4）薄层色谱中应显与连翘相对应的斑点	显相对应的斑点	符合规定
[检查]			
相对密度：	应不低于 1.07	1.13	符合规定
PH 值：	应为 4.5—6.0	5.3	符合规定
装量差异：	应符合规定	符合规定	符合规定
[微生物限度]	应符合规定	符合规定	符合规定
[含量]	含栀子苷每 1ml 不得少于 0.70mg	1.78mg/ml	符合规定

结　论	本品按国家食品药品监督管理局药品标准 YBZ05062017 检验，结果符合规定。
备　注	

（印章：邯郸制药股份有限公司 检验专用章 130401[illegible]040167）

检验负责人 庞秋超　　　　复核人 赵婷　　　　报告人 江思雨

邯郸制药股份有限公司

产品检验报告书

报告书编号：

检品名称	小儿风热清口服液（模拟剂）	批　　号	XE23002	收检日期	2023年08月17日
包　　装	玻璃瓶及纸盒	生产车间	综合制剂车间	报告日期	2023年08月23日
规　　格	10毫升×6支×6盒	检验目的	出厂检验	产　　量	9000支
检品数量	40支	检验项目	全检	送 检 人	牛肖翠
检验依据	国家食品药品监督管理局药品标准 YBZ05062017				

检验项目	标准规定	检验数据	项目结论
[检查]			
装量差异：	应符合规定	符合规定	符合规定
[微生物限度]	应符合规定	符合规定	符合规定

邯郸制药股份有限公司
检验专用章
1304010040167

结　论　本品按国家食品药品监督管理局药品标准 YBZ05062017 检验，结果符合规定。

备　注

检验负责人 [illegible]　　　复核人 [illegible]　　　报告人 [illegible]

附表：01-050-1　　文件编号：II01-050（2016）D

邯郸制药股份有限公司

产品检验报告书

报告书编号：

检品名称	磷酸奥司他韦颗粒	批　　号	6002305056	收检日期	2023 年 05 月 08 日
包　　装	复合膜	生产车间	综合制剂车间	报告日期	2023 年 05 月 14 日
规　　格	15 毫克/袋	检验目的	出厂检验	产　　量	15000 袋
检品数量	30 袋	检验项目	全检	送 检 人	牛肖翠
检验依据	《中国药典》2020 年版四部通则				

检验项目	标准规定	检验数据	项目结论
［检查］			
水分：	不得超过 8.0%	0.1%	符合规定
装量差异：	应符合规定	符合规定	符合规定
［微生物限度］	应符合规定	符合规定	符合规定

邯郸制药股份有限公司 检验专用章 1304010040167

结　论	本品按《中国药典》2020 年版四部通则检验，结果符合规定。
备　注	

检验负责人 庞利超　　复核人 赵婷　　报告人 江思雨

附表：01-050-1

2017 版 01
文件编号：II01-050（2016）D

邯郸制药股份有限公司

产品检验报告书

报告书编号：

检品名称	磷酸奥司他韦颗粒（模拟剂）	批　　号	M6002305056	收检日期	2023 年 08 月 18 日
包　　装	复合膜	生产车间	综合制剂车间	报告日期	2023 年 08 月 24 日
规　　格	15 毫克/袋	检验目的	出厂检验	产　　量	15600 袋
检品数量	30 袋	检验项目	全检	送 检 人	牛肖翠
检验依据	《中国药典》2020 年版四部通则				

检验项目	标准规定	检验数据	项目结论
[检查]			
水分：	不得超过 8.0%	0.04%	符合规定
装量差异：	应符合规定	符合规定	符合规定
[微生物限度]	应符合规定	符合规定	符合规定

邯郸制药股份有限公司 检验专用章 1304010040167

结　论	本品按《中国药典》2020 年版四部通则检验，结果符合规定。
备　注	

检验负责人 庞秋超　　复核人 赵婷　　报告人 江思雨
